# Early Combined B-Cell Depletion and BTK Inhibition Reduced TLS-like Structures and Relapse in PLP_139–151_-Induced EAE

**DOI:** 10.3390/ijms27125439

**Published:** 2026-06-16

**Authors:** Xiujuan Lang, Lin Fu, Feifei Tang, Miao Hao, Jiurong Liu, Zhengyi Chen, Wei Huang, Yue Sun, Yanting Meng, Yanping Wang, Yumei Liu, Xijun Liu, Bo Sun, Hulun Li

**Affiliations:** 1 Department of Neurobiology, School of Basic Medical Sciences, Harbin Medical University, Harbin 150081, China; langxiujuan11@163.com (X.L.); fu19844590661@163.com (L.F.); 2020173001@hrbmu.edu.cn (F.T.); 15245700320@163.com (M.H.); liujiurong2001@163.com (J.L.); czyhmu0810@163.com (Z.C.); huangweihmu@163.com (W.H.); mengyanting2023@163.com (Y.M.); wangy.p0614@hotmail.com (Y.W.); liuyumei@hrbmu.edu.cn (Y.L.); lxj@hrbmu.edu.cn (X.L.); 2 The Key Laboratory of Preservation of Human Genetic Resources and Disease Control in China, Harbin Medical University, Ministry of Education, Harbin 150081, China; 3 Center for Endemic Disease Control, Chinese Center for Disease Control and Prevention, Harbin Medical University, Harbin 150081, China; sunyuehmu@163.com

**Keywords:** multiple sclerosis, tertiary lymphoid structures, anti-CD20, evobrutinib

## Abstract

B-cell-depleting therapies have revolutionized multiple sclerosis (MS) treatment, yet relapses persist in some patients—suggesting additional pathogenic drivers beyond peripheral B cells. Tertiary lymphoid structures (TLS) are extensively documented in progressive MS at autopsy, but whether their formation begins during the relapsing-remitting phase and how they evolve during the transition to progression remain undefined. Here, using the relapsing-remitting PLP_139–151_-induced EAE model, we uncover that TLS-like structures form in the subventricular zone during relapse, once established, persist through remission as niches containing both B cells and persistently activated microglia. Neither B-cell depletion alone nor BTK inhibition alone fully prevents relapse. Strikingly, early combined B-cell depletion and BTK inhibition virtually abolishes TLS-like structure formation and may effectively prevent complete disease relapse in this model. By contrast, late initiation of the same combination fails to resolve existing TLS-like structures or prevent relapse, although it attenuates disease severity. These data indicate that established TLS-like structures may represent treatment-resistant compartments, and that both B cells and microglia may be crucial during early formation for sustaining their disease relapse-driving activity. Our study confirms that TLS-like structures may be a key factor driving the compartmentalization of central nervous system inflammation, points to a potentially narrow therapeutic window for intervention, and proposes that early combined B-cell depletion and BTK inhibition may represent a promising strategy worthy of further investigation.

## 1. Introduction

MS is an autoimmune disease characterized by inflammatory demyelination and neurodegeneration in the central nervous system (CNS). It is one of the leading causes of non-traumatic neurological disability among young and middle-aged adults worldwide [1,2]. According to the clinical course, MS can be classified into relapsing-remitting type (RRMS), primary progressive type (PPMS), and secondary progressive type (SPMS), etc. [3]. Approximately 85% of patients initially present with RRMS [4]. As the disease progresses, 50% to 60% of RRMS patients will transform into SPMS within 10 to 20 years. This phase is characterized by the progressive accumulation of neurological damage, leading to a gradual, irreversible progression of disability without distinct remission [4,5,6]. The persistent activation of microglia in the CNS, mitochondrial dysfunction, iron deposition, and oxidative stress collectively form the pathological basis of SPMS, which poses the most significant challenge in current clinical treatment [7,8,9]. Anti-B cell therapy can effectively alleviate the relapse of RRMS [8], yet it demonstrates no therapeutic efficacy in progressive MS [10,11]. These findings from clinical data and fundamental research indicate that there are still many unknown aspects in our understanding of the pathogenesis of MS.

In the late 1970s, postmortem examinations of MS patients revealed the presence of TLS within the brains of those with SPMS. These structures form abnormally in the meninges and around parenchymal lesions [12]. TLS are aggregations of immune cells similar to secondary lymphoid organs, comprising B cell zones, T cell zones, follicular dendritic cells, and a network of stromal cells [13]. Some early studies have found that the presence of TLS is significantly associated with an earlier onset age, a faster rate of disability progression, and a higher mortality rate [12,14]. However, since its discovery, TLS have not received much attention in research. This is largely due to the fact that TLS are difficult to detect with current clinical examination methods, and the autopsy samples of MS patients are extremely scarce. It is still unclear about the role of TLS in the pathogenesis of MS, as well as the relationship between TLS and disease relapse and progression. What does the presence of TLS in the brain truly signify for the disease progression and treatment of MS patients? Could the TLS be a structure we have long overlooked but that plays a crucial role in the pathogenesis of MS?

For this purpose, this study established an experimental autoimmune encephalomyelitis (EAE) model of MS by immunizing SJL/J mice with the peptide segment PLP_139–151_ (PLP_139–151_-EAE_SJL/J_) in order to explore the dynamics of TLS formation throughout the disease course, the characteristics of the pathogenesis of EAE with the presence of TLS in the brain, as well as their relationship with EAE relapse. The results showed that in the PLP_139–151_-EAE_SJL/J_ model mice, the presence or absence of TLS-like structures is closely associated with disease relapse. Once TLS-like structures were formed, they would not disappear even when the disease was in remission. In addition to abundant B cells, TLS-like structures also contain small numbers of T cells, dendritic cells, and persistently activated microglia. The activated microglia in TLS-like structures, rather than B cells, may determine the severity of EAE relapse. Before the formation of TLS-like structures, eliminating B cells coupled with inhibiting activated microglia can prevent disease relapse. However, after the formation of TLS-like structures, this therapeutic approach can mitigate relapse severity but does not fully protect against disease relapse. These data suggest that TLS-like structures are associated with relapse and may contribute to compartmentalized CNS inflammation in this EAE model. Our study may underscore the importance of early combination therapy and TLS-based patient stratification for improving outcomes in relapsing neuroinflammatory diseases.

## 2. Results

### 2.1. Relapse-Associated Spinal Cord Inflammation and Demyelination Are Fully Reversed During Remission in PLP_139–151_-EAE_SJL/J_ Model Mice

**Figure 1:**
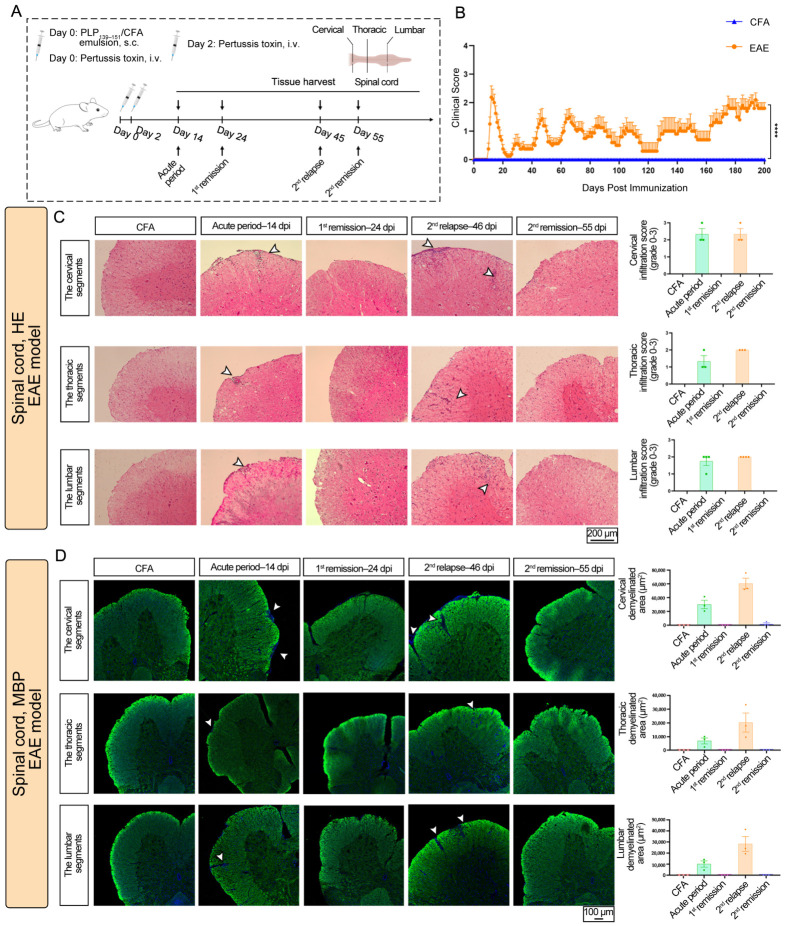
Detection of inflammatory cell infiltration and demyelination in the spinal cords of the PLP_139–151_-EAE_SJL/J_ mice at different stages of the disease. (**A**) Schematic diagram of EAE model induction and sample collection. (**B**) Clinical disease course. (EAE, *n* = 8; CFA, *n* = 8). Mean values ± SEM. Mixed–effects analysis, multiple comparisons; 95% CI of difference (0.6803 to 1.205). **** *p* < 0.0001. (**C**,**D**) Pathological analyses of cervical, thoracic, and lumbar spinal cord tissues during the acute episode, the corresponding remission period, the second relapse, and the subsequent remission period. (**C**) H&E staining and inflammatory infiltration scoring. The white arrowhead indicates the inflammatory cell infiltration area. Scoring criteria: Grade 0, minimal or few scattered inflammatory cells; Grade 1, sparse, scattered cells without confluent foci; Grade 2, moderate cells with small foci or continuous strands; Grade 3, dense and extensive infiltration, often forming large confluent areas. For the scoring method, one frozen section per spinal cord segment per mouse (CFA, *n* = 3; acute period, *n* = 3 (for lumbar, *n* = 4); 1st remission, *n* = 3; 2nd relapse, *n* = 3 (for lumbar, *n* = 4); 2nd remission, *n* = 3) was examined. Each inflammatory focus was scored individually; the average score per segment per mouse was calculated, followed by the group mean for each segment. Mean values ± SEM. Kruskal–Wallis test with Dunn’s post hoc comparison. The scale bars represent 200 μm. (**D**) MBP staining and quantification of demyelinated areas. The white arrowhead indicates the demyelinated area. For each group (CFA, *n* = 3; acute period, *n* = 3; 1st remission, *n* = 3; 2nd relapse, *n* = 3; 2nd remission, *n* = 3), one frozen section from each spinal segment (cervical, thoracic, lumbar) was analyzed. The area of each demyelinated lesion was measured using ImageJ software 1.54, and the sum of lesion areas per section was calculated as the demyelinated area for that segment in that mouse. The demyelinated areas from the same segment across all mice in a group were averaged to obtain the group mean for each segment. Mean values ± SEM. Kruskal–Wallis test with Dunn’s post hoc comparison. The scale bars represent 100 μm. s.c., subcutaneous injection; i.v., intravenous injection. A total of 33 mice were used.

We synthesized the PLP_139–151_ peptide (HSFGKWLGHPDKF) and established an EAE animal model in SJL/J mice using a two-point subcutaneous immunization method on the back. Mice were randomly divided into two groups: the EAE group was immunized with PLP_139–151_ peptide emulsified in complete Freund’s adjuvant (CFA), while the control group received an emulsion of PBS and CFA. Clinical scores were recorded after disease onset in mice, with a maximum monitoring period of 200 days, and pathological examinations were performed at the corresponding time points (Figure 1A). The PLP_139–151_-EAE_SJL/J_ model mice exhibit a relapsing-remitting disease course. Since the initial acute episode is triggered by active immunization and differs from the subsequent spontaneous relapse in its pathogenesis, we define the first clinical episode as the “acute phase” and all following episodes as “relapses”. During the remission period following the acute episode, the motor function of mice showed a recovery of over 50%. However, the extent of recovery was significantly reduced during the remission period following a second relapse (only about 29% recovered), and after the third relapse, the remission period of the disease became less pronounced. The mice exhibited a persistent impairment in motor function, characterized by bradykinesia, postural rotation with a tendency to circle continuously to one side, and an overall reduction in activity levels (Table 1). In summary, the PLP_139–151_-EAE_SJL/J_ model mice exhibit a spontaneous relapsing-remitting disease course, with recurrent episodes occurring at approximately 20–day intervals. As the number of relapses increases, the degree of recovery during remission periods declines gradually (Figure 1B).

Next, we performed histopathological examinations at different stages of EAE. Given that the initial relapse around 27 days post-immunization (dpi) is characterized by low severity, with a clinical score of approximately 1 point, and is not highly representative, we selected four time points for the subsequent histopathological examinations: the peak of the first acute episode (around 14 dpi), the remission of the acute episode (around 24 dpi), the peak of the second relapse (around 46 dpi), and the remission of the second relapse (around 55 dpi). First, we examined the infiltration of lymphocytes and the degree of demyelination within the spinal cord. Three representative spinal cord segments were selected: cervical, thoracic, and lumbar. The results showed that during the peak period of the disease (whether during the acute episode or the relapse), all three segments of the mouse spinal cord exhibited marked lymphocyte infiltration. In contrast, this inflammatory infiltration diminished dramatically in the remission phase (Figure 1C, Supplementary Figure S1A). The demyelination results mirrored the trend of lymphocyte infiltration. During the peak stages of both the acute episode and the second relapse, obvious demyelination was observed in all three spinal cord segments. During the corresponding remission periods of the two disease episodes, no obvious demyelination was detected in any segment, indicating that the myelin sheaths undergo effective repair during remission (Figure 1D, Supplementary Figure S1B).

### 2.2. PLP_139–151_-EAE_SJL/J_ Model Mice Exhibit TLS-like Structure Formation in the Brain After the Acute Episode

**Figure 2:**
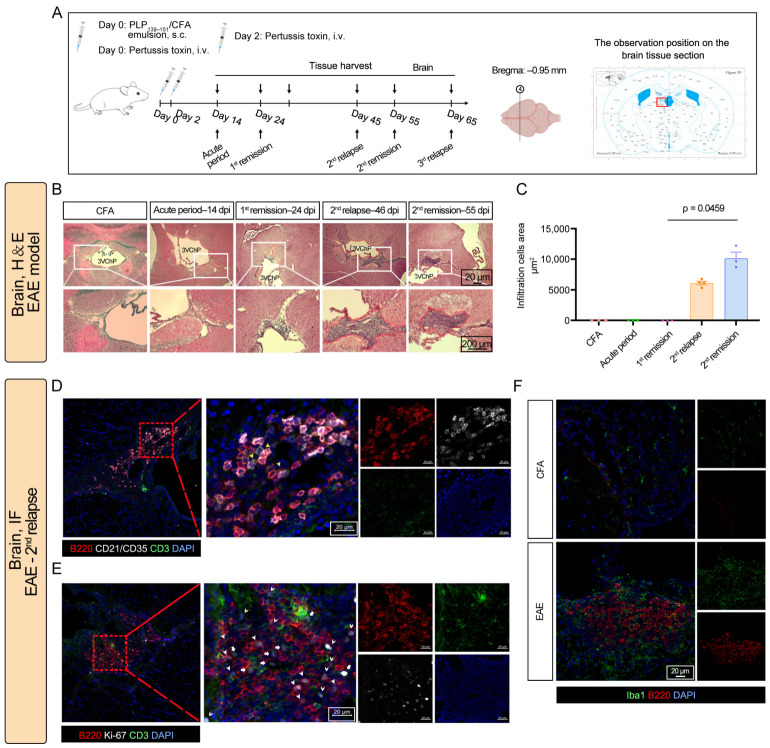
Detection of inflammatory cell infiltration in brain tissues of the PLP_139–151_-EAE_SJL/J_ mice at different stages of the disease. (**A**) Schematic of EAE model induction and sample collection, with a panel on the right depicting the analysis regions (anatomical reference was based on the Mouse Brain Library Atlas and Paxinos and Franklin’s The Mouse Brain in Stereotaxic Coordinates). (**B**) H&E staining of the brain tissues in EAE model mice during the acute episode, the corresponding remission period, the second relapse, and the subsequent remission period. In the upper panel, the red dashed box demarcates the choroid plexus in the third ventricle (3VChP), with the area within the white rectangle shown at higher magnification. In the lower panel, the red outline indicates the region of cellular infiltration into the brain parenchyma. The scale bars represent 20 μm and 200 μm. (**C**) Quantification of inflammatory cell infiltration areas in mouse brains at the indicated disease stages in Figure 2B. For each group (CFA, *n* = 3; acute period, *n* = 3; 1st remission, *n* = 3; 2nd relapse, *n* = 4; 2nd remission, *n* = 3), one brain section per mouse (region shown in (**A**)) was analyzed. Infiltration areas were measured using ImageJ software 1.54, and group means were calculated. Mean values ± SEM. Kruskal–Wallis test with Dunn’s post hoc comparison. (**D**–**F**) The tissue section was selected from the region shown in (**A**) for multiplex immunofluorescence detection in the brain of mice in the second relapse. For (**D**) B220 (red), CD3 (green), CD21/CD35 (gray), and DAPI (blue). Scale bars: 20 μm. Yellow triangular arrows indicate follicular dendritic cells. For (**E**) B220 (red), CD3 (green), Ki-67 (gray), and DAPI (blue). Scale bars: 20 μm. White arrows indicate proliferating T cells. White triangular arrows indicate proliferating B cells. V-shaped arrows indicate proliferating cells. For (**F**), B220 (red), Iba1 (green), and DAPI (blue). Scale bars: 20 μm. A total of 20 mice were used.

Subsequently, at the aforementioned four time points, we also performed H&E staining and demyelination assessment on the brains of the EAE mice. Serial coronal sections of the mouse brain tissue were prepared, and H&E staining was conducted on nine sections selected from the rostral to the caudal end. During the peak of the acute episode, no significant lymphocyte infiltration was detected in the brain (the term “lymphocyte infiltration” here specifically refers to cells that have invaded the brain parenchyma, excluding immune cells normally residing in the choroid plexus (ChP)) (Supplementary Figure S2). During the peak period of the second relapse, obvious inflammatory cell infiltration was observed in the subventricular zone (SVZ) of the third and lateral ventricles. Notably, these infiltrating cells did not dissipate during the subsequent remission period (Supplementary Figure S2). Since we found inflammatory cell infiltration only in the SVZ region, for demyelination detection, we selected the layer where inflammatory cell infiltration was detected in H&E staining. In addition to the SVZ region, three randomly chosen locations were also examined. We observed no obvious demyelination in the mouse brain during the acute episode or the subsequent remission phase. However, at the peak of the second relapse, myelin loss was evident in the SVZ, accompanied by a looser organization of the surrounding nerve fibres compared to the controls. Notably, this structural looseness persisted into the following remission phase. It is unclear whether the myelin loss seen during relapse is caused by the physical occupation of space by infiltrating and aggregating inflammatory cells (Supplementary Figure S3). Furthermore, as the disease progressed, the area occupied by these cell aggregations in the SVZ progressively expanded (Figure 2A–C).

In order to further identify the specific cell types comprising the inflammatory aggregates within the brain, we used immunofluorescence to label and identify the cell clusters that aggregated within the brain during the peak of the second relapse. The results showed that in this inflammatory structure, there were B cells (B220^+^), T cells (CD3^+^), microglia cells (Iba1^+^), and the majority of CD21/CD35 expression was confined to B cells; however, sporadic CD21/CD35^+^ cells were also observed among non-B cells (which might be follicular dendritic cells [15]) (Figure 2D–F). A few B cells and T cells are in a proliferating state, and there are also some non-lymphoid cells proliferating (Figure 2E). The microglia in the aggregated cell clusters gather together in an activated state, forming a mesh-like structure (Figure 2F). To further test the character of the aggregation of lymphoid cells, we further stained GL-7, FDC-M1, and CXCL13 expression in it. And results showed that these specific TLS markers were also positive in the B zone (Supplementary Figure S4A–C). These results further showed that the lymphocytes aggregation was a TLS-like structure, including the cell types, pattern of aggregation, and persistent state [16].

To further understand the formation and development of TLS-like structures, we performed immunofluorescent staining on the TLS-like structures at the acute episode peak, the first relapse peak, the second relapse peak, and the third relapse peak, using antibodies against FDC-M1 (marker of follicular dendritic cells), CD3 (marker of T cells), B220 (marker of B cells), CD11c (marker of dendritic cells), and Iba1 (marker of microglia). These results indicated that during the acute episode peak, a small number of B cells and T cells had already infiltrated the ChP in the SVZ, but their quantity was very limited, and no obvious TLS-like structures had emerged (Supplementary Figure S5). By the time of the first relapse, the TLS-like structures had essentially formed. With each relapse, the number of B cells, T cells, and other immune cells within this structure gradually increased, and activated microglia also gradually accumulated and multiplied. However, the number of DC cells was relatively small, and no mesh-like structure was observed (Supplementary Figure S5).

### 2.3. The TLS-like Structure Niche in EAE Brain Sustains Microglial Activation During Remission, Unlike the Spinal Cord

**Figure 3:**
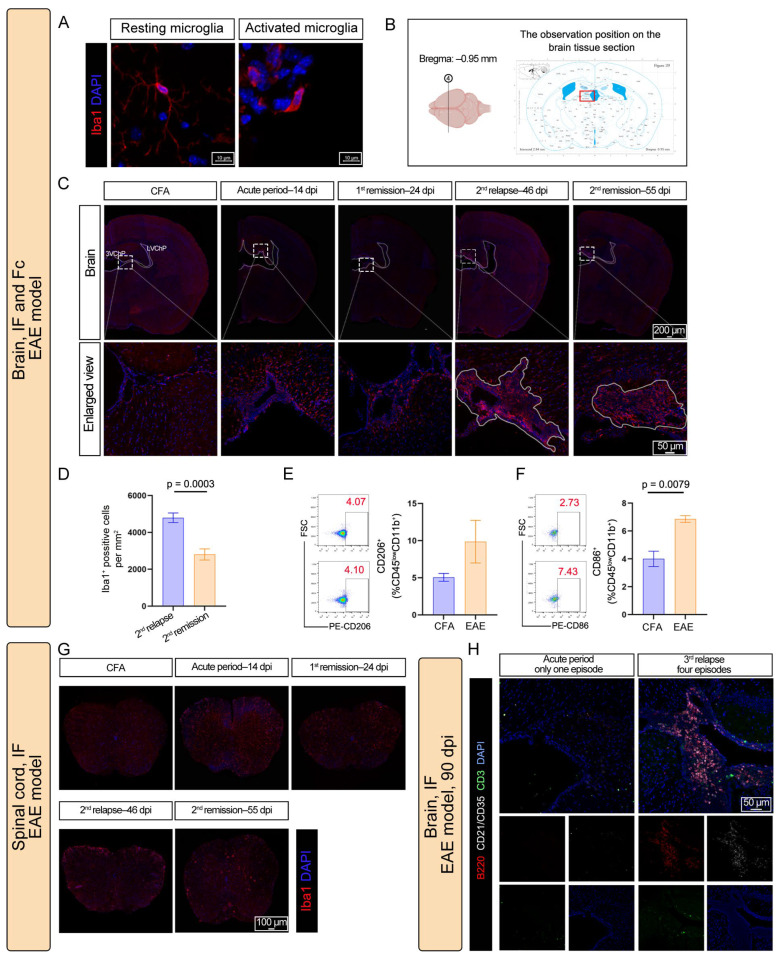
Detection of microglia in the brain and spinal cord of the PLP_139–151_-EAE_SJL/J_ mice at different stages of the disease. (**A**) Resting and activated microglia in brain tissues. Scale bars: 10 μm. (**B**) Schematic showing the region of interest for imaging in brain sections. (**C**) Iba1 immunofluorescent staining of brain tissues during the acute episode, the corresponding remission period, the second relapse, and the subsequent remission period. (**Top**): hemi-brain sections; the 3VChP area is marked by a white dashed line, and the region within the white dashed rectangle indicates the location of TLS-like structure formation, with a magnified view shown in the figure below. (**Bottom**): magnified views; The area within white line indicates Iba1^+^ activated microglia within TLS-like structures. Scale bars: 50 μm (**bottom**), 200 μm (**top**). (**D**) Quantification of Iba1^+^ cell density within TLS -like structures during the second relapse and its corresponding remission (related to (**C**)). For each group (2nd relapse, *n* = 3; 2nd remission, *n* = 3), one brain section per mouse (region shown in (**B**)) was stained. Three non-overlapping fields per section were randomly selected, and Iba1^+^ cells were counted per unit area. The average count per section was calculated from the three fields, and group means were determined. Mean values ± SEM. Mann–Whitney test. The Hodges–Lehmann estimate of the median difference was 1943 (96% CI: 1166 to 3109). (**E**,**F**) Flow cytometry analysis of the proportion of microglia in the brain of mice during secondary relapse. (**E**) Anti-inflammatory Ly6c^−^CD45^low^CD11b^+^CD206^+^ microglia (EAE, *n* = 5; CFA, *n* = 5). Mean values ± SEM. Mann–Whitney test. (**F**) Pro-inflammatory Ly6c^−^CD45^low^CD11b^+^CD86^+^ microglia (EAE, *n* = 5; CFA, *n* = 5). Mean values ± SEM. Mann–Whitney test. The Hodges–Lehmann estimate of the median difference was 3.01 (96.83% CI: 1.15 to 4.32). (**G**) Iba1 immunofluorescent staining of the spinal cord in EAE model mice during the acute episode, the corresponding remission period, the second relapse, and the subsequent remission period. Scale bars: 100 μm. (**H**) PLP_139–151_-EAE_SJL/J_ mice were established. Brain tissues from mice that experienced four episodes and those that experienced only a single episode within 90 dpi were subjected to immunofluorescent staining. B220 (red), CD3 (green), CD21/CD35 (gray) and DAPI (blue). Scale bars: 50 μm. IF, immunofluorescence; Fc, flow cytometry. A total of 18 mice were used.

Under normal conditions, microglia in the healthy brain remain in a resting state until activated by pathological stimuli (Figure 3A). During the peak phase, numerous activated microglia are recruited and aggregated within TLS-like structures, contributing to the formation of an inflammatory microenvironment in the brain [17]. Consequently, we next examined microglia activation in the SVZ of the third ventricle at different disease stages (the peak of the acute episode, the remission phase, the peak of the second relapse, and the subsequent remission phase). The results showed that during the acute episode and the subsequent remission phase, there was no significant activation of microglia in the SVZ. During the peak of the second relapse and the subsequent remission phase, activated microglia appeared within and near the TLS-like structures (Figure 3B,C). The number of activated microglia during the remission phase was significantly lower than that at the peak phase (Figure 3D). We further used flow cytometry to assess the inflammatory status of microglia in the brain. The results showed that, compared with the control CFA group, the proportion of CD86^+^ microglia (Ly6c^−^CD45^low^CD11b^+^CD86^+^) was significantly increased at the second relapse peak, while there was no statistical difference in the proportion of CD206^+^ microglia (Ly6c^−^CD45^low^CD11b^+^CD206^+^) (Figure 3E,F). The gating strategy is shown in (Supplementary Figure S6). These results indicate that microglia exhibited a predominantly pro-inflammatory activation state. To further confirm whether there were also activated microglia in other parts of the brain, we selected EAE mice at 120 dpi for long-term observation (the mice were in a state of continuous disease, and no longer exhibited an obvious remission period). We chose five coronal sections from the brains of these mice and randomly selected three fields for imaging on each section. The results revealed that activated microglia were present exclusively within and in proximity to the TLS-like structures, with no significant activation observed in other areas (Supplementary Figure S7A,B). We simultaneously assessed microglia activation in the spinal cord. In contrast to the brain, microglia in the spinal cord exhibited activation and an elevated density in both gray and white matter at the peak of the acute episode. Subsequently, during the remission phase, both the number and activation state of microglia tended to revert toward a resting state. However, during the second relapse peak, the state of microglia activation exhibited distinct characteristics compared to that observed at the peak of the acute episode. The activation and the increased population mainly occurred in the peripheral white matter (surrounding demyelinated regions), while this phenomenon was not evident in the central gray matter. During the remission period of the second relapse, the activated microglia basically returned to the resting state, and a small portion of microglia exhibited an amoeba-like morphology (Figure 3G).

The incidence of EAE in the PLP_139–151_-EAE_SJL/J_ model mice was approximately 95% in our study. Approximately 5% of mice exhibited a single episode during the whole course of the disease, meaning there was no relapse after the acute episode (Supplementary Figure S7C). At approximately 90 dpi, we examined TLS-like structures in the brains of EAE mice that had experienced relapses and those that had not. The results revealed that mice with a monophasic disease course exhibited no significant TLS-like structures in the brain, nor was infiltration of inflammatory cells detected (Figure 3H).

### 2.4. Anti-B Cell Therapy Demonstrates No Efficacy in the PLP_139–151_-EAE_SJL/J_ Model Mice

**Figure 4:**
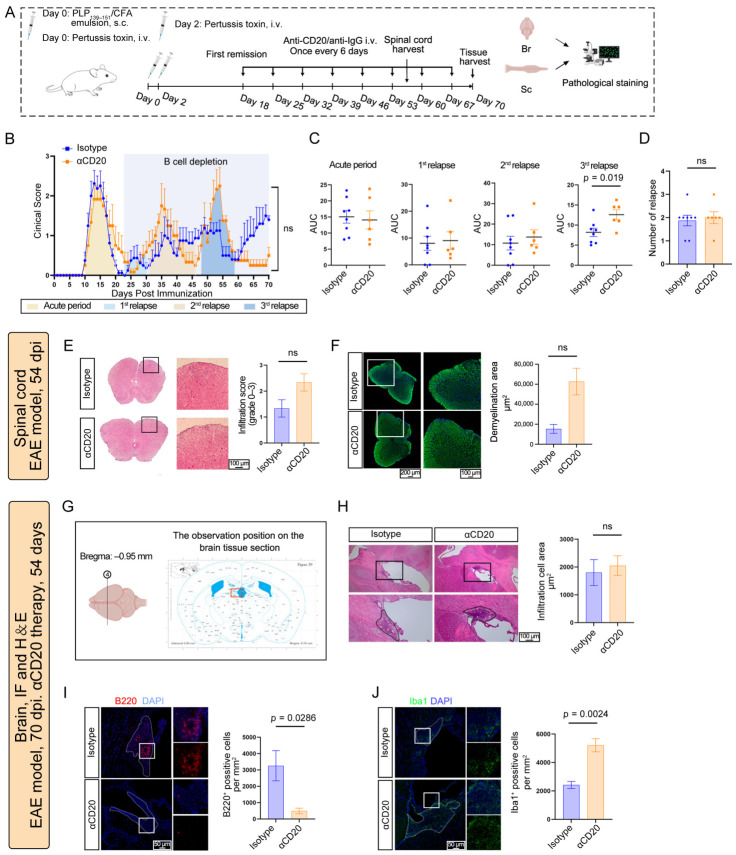
Effects of αCD20 therapy on TLS-like structures’ composition and clinical relapse in EAE mice. (**A**) Schematic diagram of αCD20 therapy in PLP_139–151_-EAE_SJL/J_ mice. (**B**) Clinical disease course of αCD20 therapy (αCD20, *n* = 6; Isotype, *n* = 8). Mixed–effects analysis. 95% CI (−0.09653 to 0.09087). (**C**) Based on the relapse/remission pattern, the disease course was divided into four stages: acute episode, first relapse, second relapse, and third relapse. Clinical disease course for each stage was analyzed by the area under the curve (AUC). Mean values ± SEM. Mann–Whitney test. The Hodges–Lehmann estimate of the median difference was 5.375 (95.74% CI: 0.7500 to 8.750). (**D**) Relapse frequency analysis. Mann–Whitney test. (**E**,**F**) Spinal cord analysis at 54 dpi in mice treated with αCD20 antibody or isotype control (αCD20, *n* = 3; Isotype, *n* = 3). (E) H&E staining and inflammatory score (αCD20, *n* = 3; Isotype, *n* = 3) (scoring as in Figure 1C). The black outline indicates the area of inflammatory cell infiltration, which corresponds to the magnified region shown on the right. Mean values ± SEM. Mann–Whitney test. Scale bars: 200 μm (overviews), 100 μm (magnified areas). (F) MBP staining and quantification of demyelination area (αCD20, *n* = 3; Isotype, *n* = 3) (method as in Figure 1D). The white outline indicates the demyelinated area, corresponding to the magnified region on the right. Mean values ± SEM. Mann–Whitney test. Scale bar: 100 μm. (**G**–**J**) Brain tissue analysis at 70 dpi. (**G**) The subsequent detection positions on the brain tissue sections. (**H**) H&E staining and quantification of inflammatory infiltration area (method as in Figure 2C). The black outlined area corresponds to the region shown in the magnified image below. Scale bar: 100 μm. αCD20, *n* = 3; Isotype, *n* = 3. Mean values ± SEM. Mann–Whitney test. (**I**) B220 immunofluorescent staining (red), DAPI (blue). White rectangle magnified at right. Scale bar: 50 μm. The TLS-like structures area was outlined with dashed box, B220^+^ cells within this region were counted, and density was calculated as cells per unit TLS-like structures area. αCD20, *n* = 4; Isotype, *n* = 4. Mean values ± SEM. Mann–Whitney test. The Hodges–Lehmann estimate of the median difference was 3289 (97.14% CI: 457.6 to 4577). (**J**) Iba1 immunofluorescent staining (green), DAPI (blue). White rectangle magnified at right; dashed box delineates TLS-like structures. Scale bar: 50 μm. Quantification of Iba1^+^ microglia density as in Figure 3D. αCD20, *n* = 3; Isotype, *n* = 3. Mean values ± SEM. Mann–Whitney test. The Hodges–Lehmann estimate of the median difference was 2861 (96% CI: 1717 to 4005). ns, not significant; IF, immunofluorescence. A total of 24 mice were used.

Anti-B cell therapy for MS has demonstrated significant clinical efficacy, yet it is not applicable to all MS patients. It has been observed that this treatment can elicit no clinical response or lead to disease exacerbation in a subset of RRMS patients [18,19,20]. To assess the therapeutic effect of B-cell depletion therapy on the PLP_139–151_-EAE_SJL/J_ model mice, female SJL/J mice weighing 16–18 g were selected to establish the EAE model. The mice were randomly divided into two groups based on their similar body weight. One group received anti-CD20 (αCD20) antibody via tail vein injection to deplete B cells, and the control group was injected with the same dose of IgG2c isotype control antibody (Figure 4A). αCD20 antibody treatment was initiated at 18 dpi, during the remission phase following the acute episode (prior to complete TLS-like structure formation), and continued until the end of the observation period (70 dpi), totalling 53 days. The administration regimen and B cell depletion efficiency are shown in Supplementary Figure S8. Throughout the entire observation period, clinical scoring revealed no significant difference in relapse frequency between the αCD20 antibody-treated and control groups. However, compared with controls, mice in the αCD20 antibody-treated group experienced a significantly more severe third relapse (Figure 4B–D, Table 2). At the peak of the third relapse (54 dpi), we assessed the extent of inflammatory cell infiltration and demyelination in the spinal cords of both groups. The results showed no significant difference in inflammatory cell infiltration and the degree of demyelination in the lumbar spinal cord between the αCD20 antibody-treated and control groups (Figure 4E,F). At the experimental endpoint (70 dpi), we examined the histological features using H&E staining and assessed the demyelination status in the SVZ of the mouse brain tissue. Given the spatial correspondence between areas of inflammatory cell infiltration and TLS-like structures in the mouse brain, we used TLS-like structures to represent the area of parenchymal inflammatory cell infiltration in this context. The results showed that αCD20 antibody intervention did not affect the formation of TLS-like structures compared to the isotype control group, and there was no difference in the area of TLS-like structures between the two groups (Figure 4G,H). Myelin staining near TLS-like structures revealed weaker signal intensity in the αCD20 antibody group (Supplementary Figure S9A). To further analyze whether the cellular components of TLS-like structures had changed, we performed immunofluorescent staining on the TLS-like structures within the brain at the end of the disease observation period (70 dpi). The results showed that in the αCD20 antibody group, the number of B cells per unit area within TLS-like structures was significantly decreased (Figure 4I), whereas the number of activated microglia per unit area was significantly increased (Figure 4J). To further clarify this observation, we assessed B cells in the brain by flow cytometry after anti-CD20 antibody treatment. The results showed that the treatment significantly reduced the proportion of B cells (Supplementary Figure S10A) and concurrently increased the proportion of microglia (Supplementary Figure S10B) in the brain.

### 2.5. Evobrutinib Inhibits Microglia Activation and Alleviates the Disease Severity but Fails to Prevent Disease Relapse

**Figure 5:**
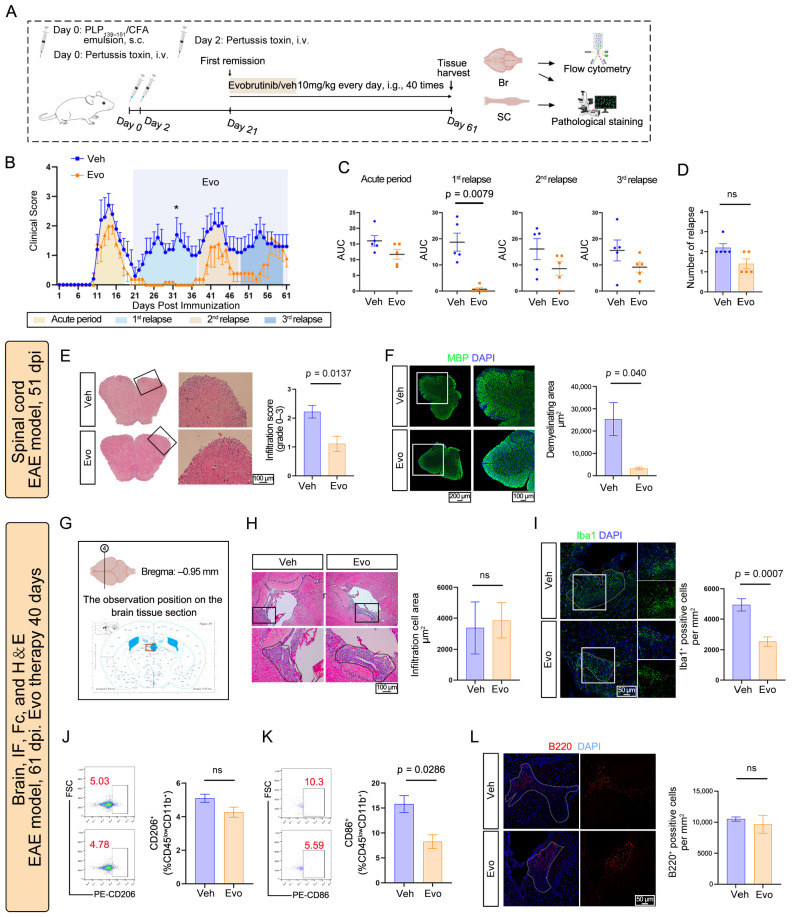
Effects of Evo therapy on clearing brain TLS-like structures and suppressing clinical relapse in EAE mice. (**A**) Schematic of the Evo treatment regimen. (**B**) Clinical disease course. Evo, *n* = 5; Veh, *n* = 5. *p* = 0.0189; Mixed–effects analysis; 95% CI (0.1777 to 1.127). (**C**) AUC of clinical scores for each disease episode in (**B**). Mean ± SEM. Mann–Whitney test. The Hodges–Lehmann estimate of the median difference was 14.5 (95.74% CI: 11 to 28.5). (**D**) Relapse frequency in (**B**). Mean ± SEM. Mann–Whitney test. (**E**,**F**) Spinal cord analysis at 51 dpi. (**E**) H&E staining and inflammatory scores (Evo, *n* = 3; Veh, *n* = 3) (scoring as in Figure 1C). The black outline indicates the area of inflammatory cell infiltration, which corresponds to the magnified region shown on the right. Mean values ± SEM. Mann–Whitney test. The Hodges–Lehmann estimate of the median difference was 1 (96% CI: 0 to 2). Scale bar: 100 μm. (**F**) MBP staining and demyelinated area quantification (Evo, *n* = 4; Veh, *n* = 4) (method as in Figure 1D). The white outline indicates the demyelinated area, corresponding to the magnified region on the right. Mean values ± SEM. Mann–Whitney test. The Hodges–Lehmann estimate of the median difference was 30,000 (97.14% CI: 6665 to 153,923). Scale bars: 200 μm (overviews), 100 μm (magnified areas). (**G**–**I**) Brain tissue analysis at 61 dpi. (**G**) The subsequent detection positions on the brain tissue sections. (**H**) H&E staining and quantification of inflammatory area (Evo, *n* = 3; Veh, *n* = 3) (method as in Figure 2C). The black outlined area corresponds to the region shown in the magnified image below. Scale bar: 100 μm. Mean values ± SEM. Mann–Whitney test. (**I**) Iba1 immunofluorescence. Iba1 (green); DAPI (blue); white dashed boxes indicate TLS-like structures. The white outlined area corresponds to the region shown in the magnified image on the right. Scale bar: 50 μm. Quantification of Iba1^+^ cell density (Evo, *n* = 3; Veh, *n* = 3) (method as in Figure 3D). Mean values ± SEM. Mann–Whitney test. The Hodges–Lehmann estimate of the median difference was 2912 (96% CI: 1165 to 3956). (**J**,**K**) Flow cytometry of brain microglia. (**J**) Proportion of anti-inflammatory Ly6c^−^CD45^low^CD11b^+^CD206^+^ microglia (Evo, *n* = 3; Veh, *n* = 3). Mean ± SEM. Mann–Whitney test. (K) Proportion of pro-inflammatory Ly6c^−^CD45^low^CD11b^+^CD86^+^ microglia (Evo, *n* = 4; Veh, *n* = 5). Mean values ± SEM. Mann–Whitney test. The Hodges–Lehmann estimate of the median difference was 6.155 (96.83% CI: 0.3 to 12.98). (**L**) B220 immunofluorescent staining and the statistics of B220^+^ B cells per unit area in the TLS-like structures of the brain tissues. The white dashed box demarcates the TLS-like structures. B220 (red), DAPI (blue). Scale bar: 50 μm. Quantification of B220^+^ B cell density (Evo, *n* = 3; Veh, *n* = 3) (as in Figure 4I). Mean values ± SEM. Mann–Whitney test. ns, not significant; i.g., oral gavage; IF, immunofluorescence; Fc, flow cytometry. A total of 26 mice were used.

The preceding results indicate that αCD20 antibody therapy failed to suppress TLS-like structure formation, reduce the frequency of EAE relapses, or ameliorate the severity of each disease episode. However, the treatment led to an increase in the number of activated microglia within TLS-like structures. To further clarify the role of microglia in EAE relapses, we employed Evobrutinib (Evo) to treat EAE mice; studies have shown that Evobrutinib can effectively suppress the expression of activation markers in microglia. Female SJL/J mice weighing 16–18 g were selected to establish the EAE model. The mice were randomly divided into two groups matched for body weight. Evobrutinib treatment was initiated at 21 dpi, during the remission phase following the acute attack, and continued until the end of the observation period (61 dpi), totalling 41 days. Mice in the Evo group received Evo by gavage, while the vehicle control group received the vehicle used to prepare Evo (i.e., DMSO and normal saline) under the same conditions. The treatment regimen is shown in Figure 5A. Over the 61–day observation period, clinical scores showed that the Evo intervention did not completely prevent EAE relapses or alleviate relapse severity, but it significantly reduced relapse frequency (Figure 5B–D, Table 3). Notably, at specific time points, we observed a reduction in disease severity; for instance, at 51 dpi, we collected the spinal cords for H&E and MBP staining. The results showed that Evo treatment significantly reduced cellular infiltration in the spinal cord. Correspondingly, MBP staining demonstrated a markedly smaller area of myelin sheath damage in the Evo-treated group (Figure 5E,F). The discrepancy between histopathological findings and clinical scores may be attributed to a temporal asynchrony in disease onset. After the disease observation period ended (61 dpi), we examined the pathological changes in the brain. The H&E staining showed that there was no difference in the TLS-like structures area between the two groups (Figure 5G,H), and no significant difference was observed in the myelin sheath damage near the TLS-like structures (Supplementary Figure S8B).

We also employed immunofluorescence to examine cellular changes within the intracranial TLS-like structures and found that, compared to the control group, the number of activated microglia per unit area within TLS-like structures was significantly lower in the Evo-treated group (Figure 5I). To further quantitatively analyze the state of intracerebral microglia, we isolated microglia from the brain and analyzed the proportions of CD86^+^ and CD206^+^ microglia using flow cytometry. The results showed that Evo significantly reduced the proportion of CD86^+^ microglia, but there was no significant difference in the proportion of CD206^+^ microglia (Figure 5J,K). Notably, the number of B cells within TLS-like structures was not significantly altered by Evo treatment (Figure 5L).

### 2.6. Combined Treatment with αCD20 Antibody and Evo Inhibits TLS-like Structure Formation and Prevents Disease Relapse

**Figure 6:**
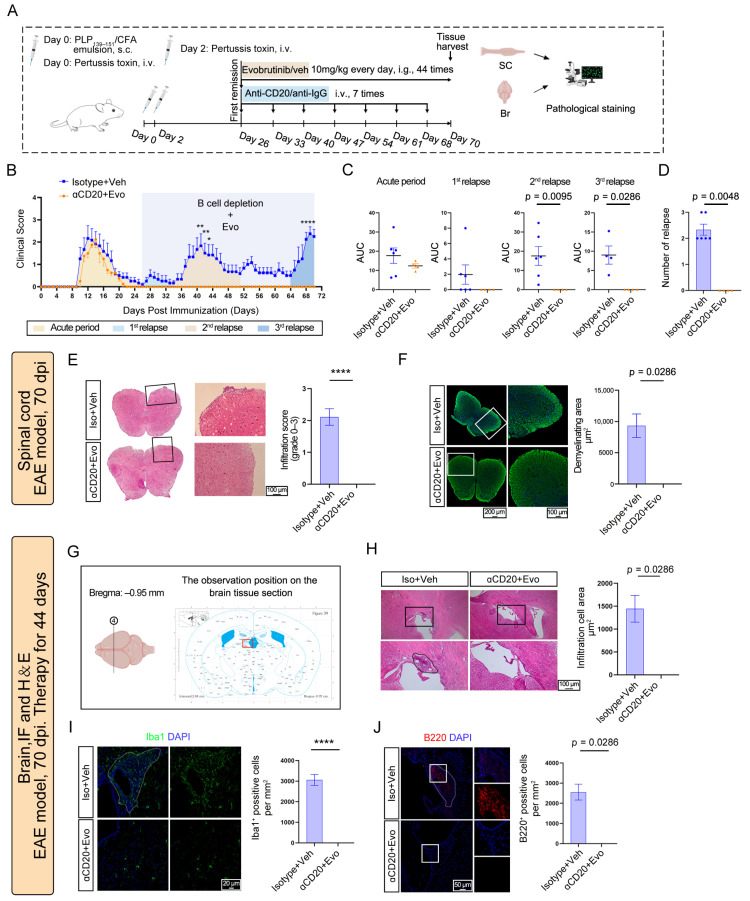
Effects of αCD20 and Evo therapy on clearing TLS-like structures in the brain and suppressing clinical relapse of EAE mice. (**A**) Schematic diagram of the combined therapy regimen in PLP_139–151_-EAE_SJL/J_ mice. (**B**) Clinical disease course of EAE mice treated with combined therapy or vehicle control. αCD20 + Evo, *n* = 4; Isotype + Evo, *n* = 6. * *p* < 0.05, ** *p* < 0.01, **** *p* < 0.0001; Mixed–effects analysis, Multiple comparisons; 95% CI of difference(0.1508 to 1.150). (**C**) AUC of clinical scores at each disease episode in (**B**). Mean values ± SEM. Mann–Whitney test. (**D**) Relapse frequency in (**B**). Mean values ± SEM. Mann–Whitney test. The Hodges–Lehmann estimate of the median difference was 2 (96.19% CI: 2 to 3). **** *p* < 0.0001. (**E**,**F**) Spinal cord tissues were collected at 70 dpi. (**E**) H&E staining and inflammatory scores (αCD20 + Evo, *n* = 3; Isotype + Evo, *n* = 3) (scoring as in Figure 1C). The black outline indicates the area of inflammatory cell infiltration, which corresponds to the magnified region shown on the right. Scale bar: 100 μm. Mean values ± SEM. Mann–Whitney test. The Hodges–Lehmann estimate of the median difference was 2 (96% CI: 1 to 3). **** *p* < 0.0001. (**F**) MBP staining and demyelination area quantification (method as in Figure 1D). The white outline indicates the demyelinated area, corresponding to the magnified region on the right. Scale bars: 200 μm (overviews), 100 μm (magnified areas). Mean values ± SEM. (αCD20 + Evo, *n* = 3; Isotype + Evo, *n* = 3), Mann–Whitney test. The Hodges–Lehmann estimate of the median difference was 8127 (94.29% CI: 6833 to 13,015). (**G**–**J**) Brain tissues collected at 70 dpi. (G) The subsequent detection positions on the brain tissue sections. (**H**) H&E staining and quantification of inflammatory cell infiltration area (αCD20 + Evo, *n* = 4; Isotype + Evo, *n* = 3) (method as in Figure 2C). The black outlined area corresponds to the region shown in the magnified image below. Scale bar: 100 μm. Mean values ± SEM. Mann–Whitney test. The Hodges–Lehmann estimate of the median difference was 1183(94.25% CI: 1122 to 2028). (**I**) Iba1 immunofluorescent staining and quantification of Iba1^+^ microglial density within TLS-like structures (αCD20 + Evo, *n* = 3; Isotype + Evo, *n* = 3) (method as in Figure 3D). The white dashed box outlines the TLS-like structures. Iba1 (green), DAPI (blue). Scale bar: 20 μm. Mean values ± SEM. Mann–Whitney test. The Hodges–Lehmann estimate of the median difference was 3047 (96% CI: 2277 to 3985). **** *p* < 0.0001. (**J**) B220 immunofluorescent staining and quantification of B220^+^ B cell density within TLS-like structures (αCD20 + Evo, *n* = 4; Isotype + Evo, *n* = 4) (method as in Figure 4I). White rectangle magnified at right; white dashed box outlines TLS-like structures. B220 (red), DAPI (blue). Scale bar: 50 μm. Mean values ± SEM. Mann–Whitney test. The Hodges–Lehmann estimate of the median difference was 3191 (97.14% CI: 2455 to 3839). IF, immunofluorescence. A total of 20 mice were used.

The results above indicate that neither depletion of B cells nor intervention of microglia and B cells with Evo can suppress disease relapse or block TLS-like structure formation. Therefore, we attempted a combined intervention using αCD20 antibody and Evo (hereafter referred to as combination therapy) to examine the impact on EAE progression. Female SJL/J mice weighing 16–18 g were selected to establish the EAE model. After remission of the acute episode, the mice were randomly divided into two groups matched for body weight: one group received αCD20 and Evo, and the control group received the same dose of IgG2c and vehicle. Combination therapy was initiated at 26 dpi, during the remission phase following the acute attack, and continued until the end of the observation period (70 dpi), totalling 45 days. The treatment regimen is shown in Figure 6A. Throughout the 70-day observation period, the clinical scores showed that the mice in the combination therapy group remained relapse-free (Figure 6B–D, Table 4). At the end of the observation period, pathological examination of the spinal cord revealed no obvious infiltration of inflammatory cells or demyelination in the combination therapy group (Figure 6E,F). Surprisingly, no TLS-like structures were detected in the brain, and no myelin damage was observed by MBP staining in the combination therapy group (Figure 6G,H, Supplementary Figure S8C). We further confirmed the status of B cells and activated microglia in the brain using immunofluorescence staining. The results showed that in the combination therapy group, no B cells or activated microglia were detected in the periventricular region (Figure 6I,J). To further clarify this observation, we assessed B cells in the brain by flow cytometry after combination treatment. The results showed that the treatment significantly reduced the proportion of B cells (Supplementary Figure S11A,B) and microglia (Supplementary Figure S11C,D) in the brain.

### 2.7. Combined Intervention Administered After TLS-like Structure Formation Failed to Effectively Eliminate Existing TLS-like Structures

**Figure 7:**
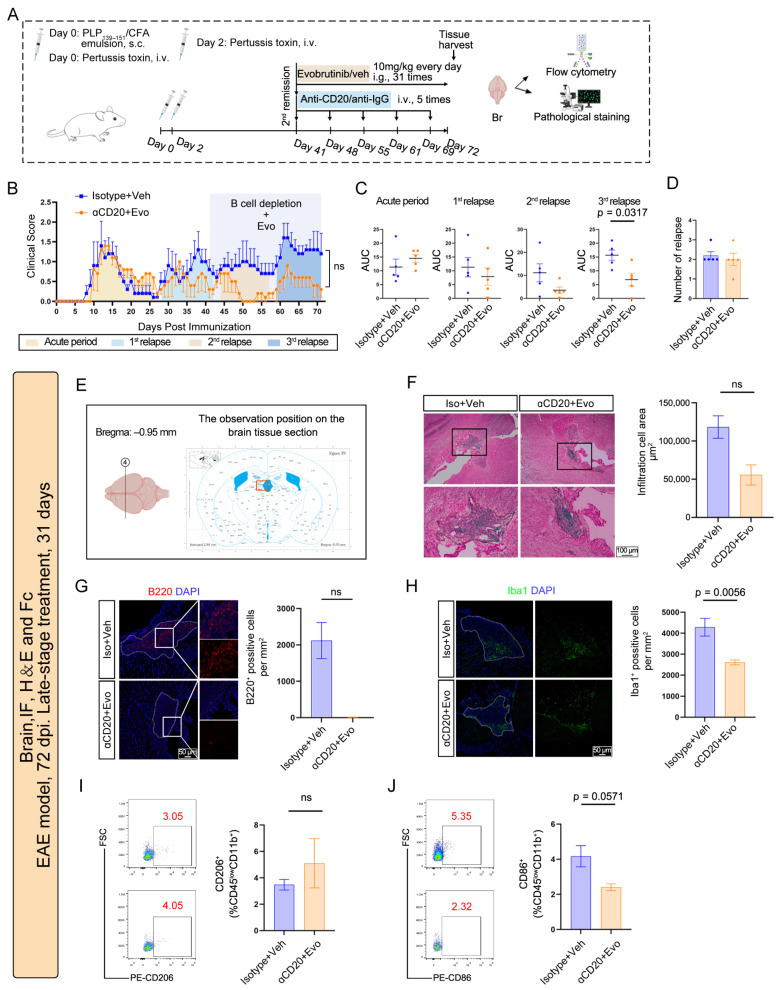
Effects of late combined therapy on clearing TLS-like structures in the brain and suppressing clinical relapse of EAE mice. (**A**) Schematic diagram of the late combined therapy in EAE model mice. (**B**) Clinical disease course of EAE mice receiving late combined therapy. Late-αCD20 + Evo, *n* = 5; late-Isotype + Veh, *n* = 5. 2 way ANOVA Multiple comparisons; 95% CI of difference(−0.7453 to 0.2342). (**C**) AUC of clinical scores at each episode stage in EAE mice treated with late combined therapy and the control vehicle in (**B**). Mean values ± SEM. Mann–Whitney test. The Hodges–Lehmann estimate of the median difference was 8.75 (96.83% CI: 2 to 16.5). (**D**) Statistical analysis of relapse frequency in (**B**). Mean values ± SEM. Mann–Whitney test. (**E**–**J**) The brain tissues of mice treated with the late combined therapy and the control vehicle were collected at 72 dpi. (**E**) The subsequent detection positions on the brain tissue sections. (**F**) H&E staining and magnified views of inflammatory infiltration areas (Late-αCD20 + Evo, *n* = 3; late-Isotype + Veh, *n* = 4) (quantified as in Figure 2C). The black outlined area corresponds to the region shown in the magnified image below. Scale bar: 100 μm. Mean values ± SEM. Mann–Whitney test. The Hodges–Lehmann estimate of the median difference was 83,150 (94.29% CI: 47,103 to 119,196). (**G**) B220 immunofluorescence staining and quantification of B220^+^ B cells within TLS-like structures (Late-αCD20 + Evo, *n* = 3; late-Isotype + Veh, *n* = 4). The white rectangle (**left**) is magnified in the right panel; the white dashed box demarcates TLS-like structures. B220 (red), DAPI (blue). Scale bar: 50 μm. Quantification was performed as in Figure 4I. Mean values ± SEM. Mann–Whitney test. (**H**) Iba1 immunofluorescence staining and quantification of Iba1^+^ microglia within TLS-like structures (Late-αCD20 + Evo, *n* = 3; late-Isotype + Veh, *n* = 3). The white dashed box demarcates TLS-like structures. Iba1 (green), DAPI (blue). Scale bar: 50 μm. Quantification was performed as in Figure 3D. Mean values ± SEM. Mann–Whitney test. The Hodges–Lehmann estimate of the median difference was 1591 (96% CI: 397.8 to 2784). I to J Flow cytometric analysis of microglial phenotypes in the brain following late combined therapy and the control vehicle. (**I**) Proportion anti-inflammatory Ly6c^−^CD45^low^CD11b^+^CD206^+^ microglia(Late-αCD20 + Evo, *n* = 3; late-Isotype + Veh, *n* = 3). Mann–Whitney test. (**J**) Pro-inflammatory Ly6c^−^CD45^low^CD11b^+^CD86^+^ microglia (Late-αCD20 + Evo, *n* = 3; late-Isotype + Veh, *n* = 3). Mean values ± SEM. Mann–Whitney test. ns, not significant; IF, immunofluorescence; Fc, flow cytometry. A total of 16 mice were used.

Although combination therapy was relatively successful in treating EAE. However, the chosen treatment window, which preceded the complete establishment of TLS-like structures, was analogous to a prophylactic setting. To assess whether this approach could similarly eliminate existing TLS-like structures, we conducted combination therapy after TLS-like structures were established at 35 dpi (hereafter referred to as late combination therapy). Female SJL/J mice weighing 16–18 g were selected to establish the EAE model. At the stage of 2nd remission, the mice were randomly divided into two groups based on their similar body weight at this stage: one group received αCD20 and Evo, and the control group received the same dose of IgG2c and vehicle (Figure 7A). Late combination treatment was initiated at 41 dpi, during the remission phase following the 1st relapse, and continued until the end of the observation period (72 dpi), totalling 32 days. Following an observation of 72 days, the clinical assessment results showed that compared with the control group, the late combination therapy failed to reduce the relapse frequency. However, the severity of the third relapse was significantly reduced, and the recovery after each relapse was also better than that of the control group (Figure 7B–D, Table 5).

Late combination therapy failed to eliminate TLS-like structures in the brain, but it reduced the area of TLS-like structures (Figure 7E,F) and decreased the number of B cells and activated microglia within TLS-like structures (Figure 7G,H). Flow cytometric analysis further demonstrated that this group exhibited significantly reduced proportions of B cells (Supplementary Figure S12). No significant difference was observed in the myelin sheath damage near TLS-like structures (Supplementary Figure S8D). Flow cytometry analysis of microglia extracted from the brain showed a decreasing trend in the proportion of pro-inflammatory CD86^+^ microglia in the late combination therapy group, whereas the percentage of anti-inflammatory CD206^+^ microglia did not show a significant difference (Figure 7I,J).

## 3. Discussion

It has been over 20 years since TLS were first discovered in the brains of MS patients [21]. However, due to the extreme difficulty in obtaining samples and the scarcity of subsequent research, this structure gradually faded from the research focus in MS. For a long time, MS (regardless of subtype) has been considered to be initiated by peripherally derived pathogenic adaptive immune cells, including T cells and B cells. Consequently, therapeutic strategies have predominantly focused on modulating these two cell types [22,23,24,25,26]. In recent years, anti-B cell therapy has demonstrated highly effective therapy in the treatment of RRMS [27], which further confirmed the central role of B cells in the pathogenesis of MS. However, some patients, particularly those with progressive MS, show insensitivity to anti-B cell therapy. Furthermore, cases where clinical anti-B cell treatments exacerbate the disease have been occasionally reported [28], suggesting that our understanding of the pathogenesis of MS remains incomplete and that there are many unknown areas waiting for us to explore. What role do the TLS play in MS/EAE? Could their existence explain some of the current unknown mechanisms of MS/EAE pathogenesis?

In the EAE model established in SJL/J mice using the PLP_139–151_ peptide, TLS-like structures form spontaneously within the brain [29,30]. Therefore, we conducted in-depth research on the pathogenesis of PLP_139–151_-EAE_SJL/J_ model mice. We found that, regardless of the acute episode or relapse, different segments of the mouse spinal cord exhibited varying degrees of inflammatory cell infiltration and demyelination. During the remission period of the disease, the inflammatory cells in the spinal cord disappeared, and the myelin sheath was repaired. In contrast, the pathological changes in the brain are different from those in the spinal cord. During the acute episode of the disease, when we used H&E staining on brain tissues, no obvious inflammatory cell infiltration was observed. However, using immunofluorescence, we observed a small number of B cells, T cells, and activated microglia in the SVZ (as immunofluorescence offers greater sensitivity for cellular detection compared to H&E staining). Yet no obvious TLS-like structures had emerged at this stage. During the subsequent first relapse period, the TLS-like structures exhibited a distinct morphology. We found that the intracranial TLS-like structures do not resolve during the remission phase of the disease. Besides B cells, T cells, and DC cells, there were also a large number of activated microglia in TLS-like structures. No areas of microglia activation were found in the brain regions outside the TLS-like structures. Studies have reported that the ChP in the brain serves as the gateway for peripheral immune cells to enter the brain [31]. This might be the reason why immune cell infiltration first appears in the SVZ. The formation of TLS may be triggered by the inflammatory response in the brain caused by active immunity: the pro-inflammatory T and B cells enter the periventricular parenchyma through the ChP, and the resulting inflammatory response activates microglia [32,33], thereby initiating the formation of TLS. We did not detect the network structure formed by DC cells in TLS-like structures. Instead, a large number of activated microglia presented a stromal structure shape. It cannot be excluded that the activated microglia in TLS-like structures provide chemotactic, adhesion, and co-stimulatory signals for T and B cells.

Concurrently, we found that TLS-like structures exhibit a close association with the relapse of EAE. In a small number of mice that did not experience relapse, no TLS-like structures were observed in the brains. In contrast, TLS-like structures were found to have formed in the brains of all mice that relapsed. When we combined the drugs to eliminate TLS-like structures, the relapse in the mice was completely inhibited. Based on these results, we conclude that TLS-like structures may be the cause of disease relapse in the PLP_139–151_-EAE_SJL/J_ mice.

In order to further explore the role of TLS-like structures in the pathogenesis of EAE, we intervened in B cells and activated microglia separately. Since well-defined TLS-like structures emerge following the remission of the acute episode, we administered B-cell depletion therapy prior to their formation. We anticipated that this treatment approach would inhibit disease progression. However, the outcome was surprising; this approach not only failed to suppress disease relapse but showed a trend toward exacerbating relapse severity. These results appear to suggest that B cells play an immunomodulatory role within TLS-like structures. What is more interesting is that after the B cells are absent in the TLS-like structures, the number of activated microglia cells significantly increases. It seems that the B cells in TLS-like structures exert an inhibitory effect on the activation and proliferation of microglia.

In the subsequent intervention experiments aimed at suppressing microglia activation, we conducted examinations at the same time point to investigate the role of activated microglia in the formation of TLS-like structures. We used the BTK inhibitor-Evo to treat the EAE mice. Evo can simultaneously inhibit the activation of B cells and microglia [34,35]. However, since our aforementioned results showed that the absence of B cells had little impact on suppressing the disease progression, we propose that the primary therapeutic effect of Evo intervention is mediated through the suppression of microglia activation.

We found that Evo treatment significantly inhibited polarization towards pro-inflammatory CD86^+^ microglia. However, Evo did not suppress TLS-like structure formation or prevent disease relapse; it significantly reduced relapse frequency and disease severity, indicating that activated microglia may be involved in the mechanisms underlying disease relapse. The dosage of Evo we chose was based on a previous study [34]. However, we noticed that while Evo intervention did not completely inhibit the activation process of microglia, it did significantly reduce the total number of activated cells. How closely are TLS-like structure formation and disease relapse related to microglia? Will TLS-like structures still form if we completely eliminate microglia? Answers to these questions require the development of more effective methods for the precisely targeted ablation of microglia in the SVZ area.

Subsequently, we attempted to treat mice with a combination of αCD20 and Evo prior to the formation of TLS-like structures. Although our earlier experiments indicated that Evo alone could not completely suppress microglial activation, combining it with αCD20 resulted in no detectable TLS-like structures in the mouse brain and also prevented disease relapse. This finding redirected our attention to B cells. As both αCD20 and Evo can act on B cells. We hypothesize that while neither agent alone achieves complete B cell suppression, their combination may act synergistically to more effectively inhibit B cell function. Whether microglia engage in functional interactions with B cells within TLS-like structures, and which cell types play a pivotal role during TLS-like structure initiation, remain to be explored.

We still do not fully understand the precise processes governing TLS-like structure formation or the exact causes of EAE relapse. However, our current findings suggest that early therapeutic intervention can virtually abrogate TLS-like structures’ development in the brain. A critical translational consideration is that MS patients seen in clinical practice likely already harbor established CNS TLS-like structures. In our model, the initial intervention window preceded full TLS-like structures’ maturation. We therefore tested whether the same combined αCD20 and Evo regimen could act on fully formed TLS-like structures. The results were sobering: although late treatment modestly reduced relapse severity, it neither resolved pre-existing TLS-like structures nor provided protection from clinical relapse. This indicates that, at least in this model, once the TLS-like structures are formed, the combined approach may be ineffective at eliminating these structures.

Although we have not yet gained a comprehensive understanding of the structure and function of TLS-like structures, the cellular interaction driving relapse, or the crosstalking among B cells, T cells, and microglia within the brain. However, our study represents an early exploration of TLS-like structure formation within the brain and its potential contribution to relapses in MS. Our findings suggest that the pathogenic activity of relapse-associated TLS-like structures is closely linked to microglial responses, and the activity of microglia may serve as a critical permissive factor for relapse initiation, although the underlying regulatory mechanisms require further elucidation. While many questions remain open, this work provides a window into how TLS-like structures may participate in MS pathogenesis, thereby expanding the scope and mode by which the immune system contributes to neuroinflammation. It offers a novel research perspective for autoimmune diseases represented by refractory MS.

However, there are some limitations in our study. The secondary progressive EAE we induced could not fully reproduce human MS heterogeneity, progression, pathology, or treatment response. What is more, the exclusive use of female SJL/J mice limits the generalizability of our findings; whether similar lymphoid structures form in males or in other genetic backgrounds remains to be examined. The lymphoid aggregates we characterized reside in the SVZ, whereas the TLS-like structures most strongly implicated in human MS pathology are located in the meninges, particularly in progressive disease. What this anatomical distinction means, and what is the reason that leads TLS-like structures to appear in the brain, have not yet been established. Moreover, the lymphoid aggregations in SVZ showed the characteristics of the TLS-like structure, such as the separate clustering of B cells and T cells, the presence of proliferating B cells and T cells, GL7^+^ B cells, FDCs and CXCL13 in the B cell zone; however, we still need to comprehensively detect and confirm that the structure is TLS. We need to explore it further. We found a method to stop the formation of TLS-like structures in the brain at a certain time, but there is still a long journey to propose a directly translatable regimen. Sample sizes were modest in some experiments; therefore, these findings should be viewed as model-specific mechanistic observations. Their extension to human pathology, particularly to meningeal TLS-like structures and progressive MS, will require independent validation in additional models and in human tissue.

## 4. Materials and Methods

### 4.1. Animals

Clean-grade healthy female SJL/J mice aged 6 to 8 weeks (16–18 g) were purchased from Vital River Laboratory Animal Technology Co., Ltd. (Beijing, China). The mice are specific pathogen-free. All animals were housed under sterile conditions with controlled temperature (20 ± 2 °C) and a 12 h light/dark cycle at Harbin Medical University. They were fed a standard rodent diet and provided water ad libitum. The health status of the mice, including body weight and clinical scores, was monitored throughout the experimental period. All animal experiments were conducted in accordance with the Guide for the Care and Use of Laboratory Animals issued by the National Institutes of Health of China. Every effort was made to minimize animal suffering.

### 4.2. PLP_139–151_-EAE_SJL/J_ Model Mice Induction and Clinical Score

Female SJL/J mice were subcutaneously injected on the back with the PLP_139–151_ (Maokang Biotechnology Co., Ltd., Shanghai, China. 100 µg per mouse), emulsified in Complete Freund’s Adjuvant (CFA) containing heat-killed Mycobacterium tuberculosis H37RA (BD Biosciences, San Jose, CA, USA). On day 0 and day 2 post-immunization, each mouse received 400 ng of pertussis toxin (List Biological Laboratories, Inc., Campbell, CA, USA) in sterile saline via intravenous injection through the tail vein. Control mice were injected with an equivalent volume of phosphate-buffered saline (PBS) instead of the PLP_139–151_ peptide. For standard EAE induction experiments, mice of similar body weight (16–18 g) were randomly assigned to groups using a lottery method. An independent investigator, blinded to ear-tag codes and group identities, carried out the entire allocation procedure. Clinical scoring was performed daily, within a fixed time window of 2:00 p.m. to 4:00 p.m., by a single assessor who was blinded to the group allocation but familiar with the scoring criteria. The clinical observation periods were 200 days. Eight animals were assigned to each of the EAE group and the CFA group for clinical scoring. Mice were monitored for clinical signs of EAE and scored daily according to the following scale: 0: no clinical disease; 0.5: limp tail; 1: paralyzed tail; 1.5: diminished righting reflex; 2: unsteady gait; 2.5: unilateral hind limb paralysis; 3: bilateral hind limb paralysis; 3.5: paralysis of one hind leg; 4: forelimb paralysis; 5: death. The primary outcome of this experiment is the clinical score (using the EAE standard scoring system), and the secondary outcomes include body weight change and spinal cord pathology score. All subsequent experimental procedures in mice were evaluated based on these measures.

### 4.3. Tissue Preparation

Experimental mice were euthanized via transcardial perfusion with PBS to remove blood. The spinal cord and brain were then collected and fixed in 4% paraformaldehyde (PFA), followed by cryoprotection in 15% and 30% sucrose solution. Subsequently, the tissues were embedded in O.C.T. compound (Sakura, Tokyo, Japan) and snap-frozen in liquid nitrogen. Serial sections of the spinal cord were cut at a thickness of 6 µm using a cryostat, and serial sections of the brain were cut at a thickness of 10 µm.

### 4.4. Hematoxylin–Eosin (H&E) Staining

Frozen sections were stained using a standard H&E staining protocol. Briefly, sections were first stained with Mayer’s hematoxylin. After differentiation in acidified ethanol and bluing in ammonia solution, they were counterstained with eosin. The stained sections were then dehydrated through a graded ethanol series, cleared in xylene, and finally mounted with resinous mounting medium under glass coverslips for microscopic examination of tissue morphology and cellular detail. Images were acquired with an Olympus BX51 upright microscope (Olympus Corporation, Tokyo, Japan) equipped with a Nikon DS-U1 cooled color CCD camera (Nikon Corporation, Tokyo, Japan), and analyzed using ImageJ 1.54 (bundled with Java 8, NIH, Bethesda, MD, USA).

H&E staining, and the corresponding inflammatory infiltration score (Grade 0: Minimal or few scattered inflammatory cells. Grade 1: Sparse, scattered inflammatory cells without confluent foci. Grade 2: Moderate number of inflammatory cells, visible as small foci or continuous strands. Grade 3: Dense and extensive inflammatory cell infiltration, often forming large confluent areas). The group sizes used for histology (*n* = 3–4).

### 4.5. Immunolabeling

Tissue sections were post-fixed with 4% PFA and then blocked and permeabilized with 0.3% Triton X-100 and 10% fetal bovine serum for 1.5 h at room temperature. The sections were incubated with primary antibodies overnight at 4 °C. After washing three times with PBS containing 0.01% Tween 20, the sections were incubated with species-matched secondary antibodies, all diluted 1:500 in 10% normal fetal bovine serum, for 1 h in a humidified chamber at RT. Nuclei were counterstained with DAPI (4′,6-diamidino-2-phenylindole; 1:2000 dilution). Information regarding the immunofluorescent antibodies used in this study is detailed in Table A1. Imaging was performed using a fluorescence microscope (LSM700; Carl Zeiss, Jena, Germany), and images were analyzed with ZEN software (version 3.8, Carl Zeiss, Jena, Germany).

### 4.6. Flow Cytometry

Single-cell suspensions of mouse brain tissue were prepared using a 30% percoll solution. Suspensions from the spleen, lymph nodes, and peripheral blood were also obtained, followed by red blood cell lysis with ammonium-chloride-potassium lysing buffer. Flow cytometry was performed using fluorochrome-conjugated antibodies according to the manufacturer’s instructions. Dead cells were labeled with Fixable Viability Dye eFluor™ 780, and surface staining was carried out using the appropriate antibodies for 30 min at 4 °C. Details of the flow cytometry antibodies used in this study are listed in Table A1. Data acquisition was conducted on a FACSVerse flow cytometer (BD Biosciences, San Jose, CA, USA), and results were analyzed using FlowJo software (version 10, Ashland, OR, USA). The group sizes used for flow cytometry (*n* = 3–5).

### 4.7. Different Therapy in the PLP_139–151_-EAE_SJL/J_ Model Mice

αCD20 monotherapy: In the PLP_139–151_-EAE_SJL/J_ model, αCD20 antibody was administered intravenously via the tail vein to mice during the recovery phase following their first disease episode, at a dose of 100 µg per mouse, once per week. The control group was given anti-IgG2c antibody using the same experimental method.

Evo monotherapy: In the PLP_139–151_-EAE_SJL/J_ model, Evobrutinib was administered daily via oral gavage at a dose of 10 mg/kg body weight until the endpoint for sample collection. The control vehicle group received the vehicle used to prepare Evo (i.e., DMSO and normal saline) under the same conditions.

Combined therapy of αCD20 and Evo: The PLP_139–151_-EAE_SJL/J_ model mice received both αCD20 antibody and Evo starting from the recovery stage following their acute episode and the second clinical episode. αCD20 or anti-IgG2c was injected via the tail vein at 100 µg per mouse once weekly. Evobrutinib or vehicle was administered daily via oral gavage at a dose of 10 mg/kg body weight until the endpoint for sample collection.

The grouping method for mice is as follows: For therapeutic intervention experiments, baseline body weight varied because treatments were initiated during different remission phases. To eliminate this potential confounder, we used stratified block randomization. Mice were first ranked by body weight and grouped into blocks of 2–4 animals with the closest weights. Within each block, an independent investigator blinded to ear-tag codes and group identities randomly allocated the animals to experimental groups using a lottery method. This procedure ensured balanced baseline body weights across all groups while maintaining true randomization and allocation concealment. Clinical scoring was performed daily, within a fixed time window of 2:00 p.m. to 4:00 p.m., by a single assessor who was blinded to the group allocation but familiar with the scoring criteria. The observation periods were 53 days after αCD20 antibody monotherapy, 41 days after Evobrutinib monotherapy, 45 days after combination therapy, and 32 days after late combination therapy. For clinical scoring, *n* = 6–8 in the αCD20 antibody treatment group, *n* = 5 in the Evobrutinib treatment group, *n* = 4–6 in the combination treatment group, and *n* = 5 in the late combination treatment group.

### 4.8. Statistical Analysis

Longitudinal clinical scores, measured daily on an ordinal scale (0–5), were analyzed using a mixed–effects model with restricted maximum likelihood (REML) estimation, implemented in GraphPad Prism (version 9). The model included treatment group, time (days post-immunization), and their interaction as fixed effects, with individual mouse specified as a random effect to account for repeated measures. The covariance structure was selected based on the Akaike information criterion, and model assumptions were checked by inspection of studentised residuals for normality (Q–Q plot) and homoscedasticity; no major violations were detected. Following a significant group × time interaction, post hoc pairwise comparisons of estimated marginal means were performed at each time point with Šidák correction for multiplicity. Exact two–tailed *p* values and 95% confidence intervals for the differences are reported in the figures and results text.

For histological and flow-cytometric endpoints, all pairwise comparisons for these data were performed using the non-parametric Mann–Whitney U test. Exact two-tailed *p* values are reported together with the Hodges–Lehmann estimate of the median difference and its 95% confidence interval to quantify effect size and precision without reliance on parametric assumptions. Multiple-group comparisons were conducted using the Kruskal–Wallis test with Dunn’s post hoc test.

Exact sample sizes (*n*) for each group and analysis are provided in all figure legends and the corresponding results sections; no ranges are used. All statistical analyses were performed with GraphPad Prism (version 9). ImageJ was used for image analysis and quantification.

## 5. Conclusions

This study establishes that TLS-like structure formation represents a critical pathogenic event in MS relapse. We demonstrate that TLS-like structures form in the EAE brain during relapse, harbor persistently activated microglia, and once established, become structures that resist therapeutic intervention. The critical finding is that TLS-like structures are preventable: combined B-cell depletion and BTK inhibition abolishes TLS-like structure formation and prevents relapse only when initiated prior to TLS-like structures establishment, whereas the same therapy fails to eliminate pre-existing TLS-like structures or reduce relapse frequency. These findings identify the intervention timing as a determinant of therapeutic efficacy in TLS-like structures-targeted strategies, provide a mechanistic explanation for why some patients respond poorly to B-cell depletion alone, and establish TLS-like structures as both a potential biomarker for patient stratification and a therapeutic target. Our work underscores the importance of early combination therapy in relapsing neuroinflammatory diseases and opens new avenues for intervention strategies aimed at preventing, rather than reversing, CNS compartmentalized inflammation.

## Figures and Tables

**Table 1:** Clinical score analysis of EAE model mice.

Period	Onset Time	Last Time	Remission Time	Mean Score	Highest Score	Complete Remission Rate	Mean Remission Score
Acute episode	11.25 ± 1.23	9.38 ± 2.39	20.25 ± 1.64	1.40 ± 0.41	2.38 ± 0.86	0.75	0.13 ± 0.22
1st relapse	28.75 ± 3.11	4.75 ± 2.86	33.50 ± 2.60	1.11 ± 0.38	1.63 ± 0.65	0.50	0.38 ± 0.41
2nd relapse	42.29 ± 1.98	10.57 ± 3.29	52.86 ± 1.73	1.33 ± 0.50	1.79 ± 0.84	0.29	0.86 ± 0.83
3rd relapse	60.38 ± 1.87	13.13 ± 3.79	73.50 ± 3.32	1.26 ± 0.49	1.81 ± 0.90	0.13	0.75 ± 0.43 *

* Indicated scores that are significantly different from those during the remission phase following the acute episode. EAE, *n* = 8. * *p* = 0.0123. Mean values ± SD. Mann–Whitney test. The Hodges–Lehmann estimate of the median difference was 0.5 (95.01% CI: 0 to 1.0).

**Table 2:** Clinical score analysis of αCD20-treated EAE model mice.

Period	Treatment	Onset Time	Last Time	Remission Time	Mean Score	Highest Score	Complete Remission Rate	Mean Remission Score
Acute episode	Isotype	10.75 ± 0.43	9.38 ± 1.50	20.13 ± 2.11	1.64 ± 0.70	2.81 ± 0.80	1.00	0.00
	αCD20	11.33 ± 0.94	8.50 ± 1.50	19.83 ± 2.11	1.61 ± 0.70	2.25 ± 0.80	0.67	0.25 ± 0.38
1st relapse	Isotype	26.33 ± 2.62	3.33 ± 2.36	29.67 ± 0.47	1.08 ± 0.48	1.50 ± 0.82	0.67	0.17 ± 0.24
	αCD20	25.75 ± 2.77	3.00 ± 2.35	28.75 ± 2.17	1.03 ± 0.28	1.38 ± 0.22	0.25	0.88 ± 0.54
2nd relapse	Isotype	35.33 ± 3.94	9.83 ± 4.10	45.17 ± 1.86	1.46 ± 0.58	2.08 ± 0.98	0.20	0.90 ± 0.58
	αCD20	32.00 ± 1.26	10.67 ± 3.01	42.67 ± 2.5	1.34 ± 0.76	2.08 ± 1.16	1.00	0.00
3rd relapse	Isotype	49.25 ± 2.63	6.88 ± 3.59	56.13 ± 2.57	1.32 ± 0.42	1.69 ± 0.56	0.40	0.40 ± 0.37
	αCD20	49.67 ± 1.70	7.50 ± 2.63	57.17 ± 2.27	1.87 ± 0.81	2.67 ± 0.80 *	0.50	0.38 ± 0.41

* indicated a significant difference in the highest score between the αCD20-treated group (*n* = 6) and the control group (*n* = 8) during the 3rd relapse. * *p* = 0.035. The mean of the values ± SEM is shown. Mann–Whitney test. The Hodges–Lehmann estimate of the median difference was 1 (95.74% CI: 0 to 2).

**Table 3:** Clinical score analysis of Evo-treated EAE model mice.

Period	Treatment	Onset Time	Last Time	Remission Time	Mean Score	Highest Score	Complete Remission Rate	Mean Remission Score
Acute episode	Veh	10.60 ± 1.36	7.40 ± 1.36	18.00 ± 0.00	2.08 ± 0.26	3.10 ± 0.49	0.40	1.00 ± 0.95
	Evo	10.60 ± 0.80	7.00 ± 0.63	17.6 ± 0.69	1.63 ± 0.45	2.40 ± 0.73	0.60	0.40 ± 0.58
1st relapse	Veh	25.40 ± 3.61	12.00 ± 3.69	36 ± 7.97	1.57 ± 0.32	2.80 ± 0.40	0.20	1.10 ± 0.86
	Evo	25.00 ± 3.00	2.00 ± 1.00 **	27.00 ± 4.00	1.05 ± 0.55	0.75 ± 0.25	0.80	0.50 ± 0.50
2nd relapse	Veh	38.00 ± 0.00	9.00 ± 1.55	47.00 ± 1.55	1.74 ± 0.66	2.60 ± 1.11	0.40	1.10 ± 0.97
	Evo	39.50 ± 1.50	7.50 ± 1.50	47.00 ± 1.00	1.51 ± 0.57	2.38 ± 0.82	0.20	0.50 ± 0.61
3rd relapse	Veh	49.60 ± 1.20	8.60 ± 3.50	58.20 ± 3.60	1.74 ± 0.39	2.10 ± 0.49	0.20	1.30 ± 0.81
	Evo	52.40 ± 3.14	6.00 ± 3.03	58.40 ± 3.20	1.59 ± 0.55	2.00 ± 0.63	0.20	0.90 ± 0.58

** Indicated a significant difference in the last time between the Evo-treated group (*n* = 5) and the control group (*n* = 5) during the 1st relapse. ** *p* = 0.0079. The mean of the values ± SEM is shown. Mann–Whitney test. The Hodges–Lehmann estimate of the median difference was 12 (96.83% CI: 6 to 15).

**Table 4:** Clinical score analysis of αCD20- and Evo-treated EAE model mice.

Period	Treatment	Onset Time	Last Time	Remission Time	Mean Score	Highest Score	Complete Remission Rate	Mean Remission Score
Acute episode	Isotype + Veh	11.83 ± 3.67	10.50 ± 2.93	22.33 ± 2.56	1.59 ± 0.59	2.58 ± 0.98	0.83	0.08 ± 0.19
	αCD20 + Evo	11.00 ± 0.71	10.00 ± 0.71	21.00 ± 0.71	1.20 ± 0.19	2.13 ± 0.54	1.00	0.00
1st relapse	Isotype + Veh	27.33 ± 0.47	4.00 ± 0.82	31.33 ± 1.25	0.92 ± 0.38	1.00 ± 0.41	0.67	0.33 ± 0.47
	αCD20 + Evo	—	—	—	—	—	—	—
2nd relapse	Isotype + Veh	36.67 ± 2.36	9.83 ± 3.13	46.50 ± 2.29	1.42 ± 0.67	2.33 ± 0.85	0.50	0.67 ± 0.69
	αCD20 + Evo	—	—	—	—	—	—	—
3rd relapse	Isotype + Veh	65.00 ± 1.73	5.00 ± 1.73	70.00 ± 0.00	1.70 ± 0.43	2.50 ± 0.61	0.00	2.25 ± 0.56
	αCD20 + Evo	—	—	—	—	—	—	—

“—” means no relapse in the αCD20 and Evo-treated EAE model mice (*n* = 4), the control group mice were given IgG2c and vehicle (*n* = 6). The mean of the values ± SEM is shown.

**Table 5:** Clinical score analysis of late combination therapy in EAE model mice.

Period	Treatment	Onset Time	Last Time	Remission Time	Mean Score	Highest Score	Complete Remission Rate	Mean Remission Score
Acute episode	Isotype + Veh	12.40 ± 4.08	9.20 ± 2.23	21.60 ± 2.33	1.16 ± 0.33	1.90 ± 0.86	0.80	0.10 ± 0.20
	αCD20 + Evo	11.40 ± 1.2	13.60 ± 2.588	25.00 ± 2.00	1.05 ± 0.11	1.90 ± 0.49	0.20	0.60 ± 0.58
1st relapse	Isotype + Veh	29.60 ± 2.33	10.40 ± 3.88	40.00 ± 4.00	1.18 ± 0.45	1.90 ± 0.73	0.20	0.70 ± 0.40
	αCD20 + Evo	30.60 ± 3.56	7.00 ± 4.00	37.00 ± 4.86	1.01 ± 0.46	1.70 ± 1.03	0.60	0.20 ± 0.24
2nd relapse	Isotype + Veh	44.00 ± 0.00	10.80 ± 4.40	54.80 ± 4.40	0.94 ± 0.48	1.40 ± 0.97	0.20	0.70 ± 0.51
	αCD20 + Evo	48.60 ± 5.71	4.00 ± 2.28	52.60 ± 3.61	0.85 ± 0.53	1.10 ± 0.73	0.60	0.20 ± 0.24
3rd relapse	Isotype + Veh	59.40 ± 0.80	11.60 ± 0.80	71.00 ± 0.00	1.34 ± 0.28	2.00 ± 0.84	0.20	1.20 ± 1.03
	αCD20 + Evo	60.25 ± 1.09	9.75 ± 0.83	70.00 ± 1.00	0.87 ± 0.37	1.13 ± 0.41	0.50	0.38 ± 0.41

Clinical scores at treatment initiation during the remission period following the first relapse were compared between the αCD20 + Evo group (*n* = 5) and the control group (*n* = 5). The mean of the values ± SEM is shown.

## Data Availability

The data supporting the results reported in this article can be provided upon reasonable request.

## Supplementary Materials

The following supporting information can be downloaded at: https://www.mdpi.com/article/10.3390/ijms27125439/s1.

## Abbreviations

MS	Multiple Sclerosis
TLS	Tertiary lymphoid structures
SVZ	Subventricular zone
CNS	Central nervous system
RRMS	Relapsing-remitting multiple sclerosis
PPMS	Primary progressive multiple sclerosis
SPMS	Secondary progressive multiple sclerosis
EAE	Experimental autoimmune encephalomyelitis
ChP	Choroid plexus
αCD20	Anti-CD20
dpi	Days post-immunization
CFA	Complete freund’s adjuvant
3VChP	Choroid plexus in the third ventricle
AUC	Area under the curve
H&E	Hematoxylin–eosin

## Appendix A

**Table A1:** The antibody information used in this study.

Antibody	Manufacturer	Catalog Number
	Antibody for Flow cytometry	
PE/Cyanine7 Anti-Mouse CD19	Thermo Fisher Scientific, Waltham, MA, USA	Cat#25-0193-82
eBioscience™ Fixable Viability Dye eFluor™ 780	Thermo Fisher Scientific, Waltham, MA, USA	Cat#65-0865-18
PE/Cyanine7 Anti-Mouse CD11b	Thermo Fisher Scientific, Waltham, MA, USA	Cat#25-0112-82
APC Anti-Mouse CD86	BioLegend, San Diego, CA, USA	Cat#105012
PerCP/Cyanine5.5 Anti-Mouse CD4	Biolegend, San Diego, CA, USA	Cat#100434
PerCP/Cyanine5.5 Rat Anti-Mouse CD45	BD Biosciences, San Jose, CA, USA	Cat#557235
PE Anti-Mouse CD206	BioLegend, San Diego, CA, USA	Cat#141706
APC Anti-Mouse/Human CD11b	BioLegend, San Diego, CA, USA	Cat#101212
PE/Cyanine7 Anti-Mouse CD19	Thermo Fisher Scientific, Waltham, MA, USA	Cat#25-0193-82
BV421-LY6C	BioLegend, San Diego, CA, USA	Cat#1288031
Antibody for Immunofluorescence		
Rat Anti-Mouse CD45R/B220 (1:200)	BD Biosciences, San Jose, CA, USA	Cat#550286
Hamster Anti-Mouse CD3ε (1:20)	BioLegend, San Diego, CA, USA	Cat#100340
Rabbit Anti-Human/Mouse/Rat Ki-67 (D3B5) (1:400)	Cell Signaling, Danvers, MA, USA	Cat#9129
Alexa Fluor 594 anti-mouse CD21/CD35 (CR2/CR1)(1:200)	BioLegend, San Diego, CA, USA	Cat#123426
BD Pharmingen™ Purified Rat Anti-Mouse Follicular Dendritic Cell	BD Biosciences, San Jose, CA, USA	Cat#551320
FluoroMyelin™ Green (1:300)	eBioscience, San Diego, CA, USA	Cat#F34651
Mouse Anti-Mouse Iba1 (1:50)	Santa Cruz Biotechnology, Dallas, TX, USA	Cat#sc-32725
Donkey Anti-Mouse IgG (H + L) Highly Cross-Adsorbed Secondary Antibody, Alexa Fluor™ 488 (1:1000)	Invitrogen, Carlsbad, CA, USA	Cat#A-21202
Goat Anti-Rat IgG (H + L) Cross-Adsorbed Secondary Antibody, Alexa Fluor555 (1:500)	Invitrogen, Carlsbad, CA, USA	Cat#A-21434
Goat Anti-Armenian Hamster IgG (H + L) Highly Cross-Adsorbed Secondary Antibody, Alexa Fluor 488 (1:500)	Invitrogen, Carlsbad, CA, USA	Cat#A78963
Goat Anti-Rabbit IgG (H + L) Highly Cross-Adsorbed Secondary Antibody, Alexa Fluor Plus 647 (1:1000)	Invitrogen, Carlsbad, CA, USA	Cat#A32733TR
