# Supplementary material for: Early Combined B-Cell Depletion and BTK Inhibition Reduced TLS-like Structures and Relapse in PLP139–151-Induced EAE
Source: Int J Mol Sci. 2026 Jun 16;27(12):5439. doi: 10.3390/ijms27125439 (PMC13299241; doi:10.3390/ijms27125439)
Supplement: Supplementary file 1 [file ijms-27-05439-s001.zip › ijms-4322844-supplementary.pdf]

Supplementary Figure S1.

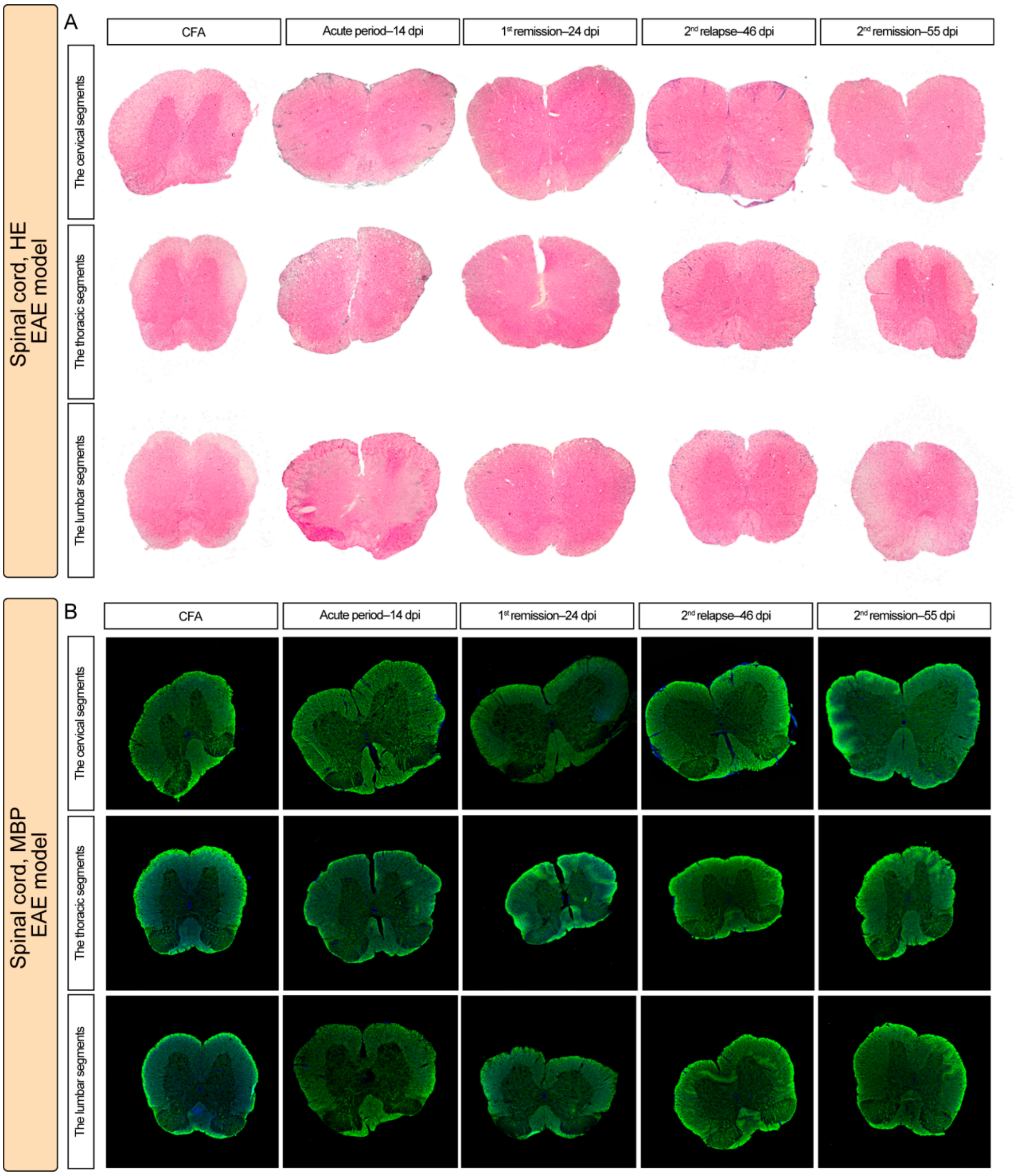

Supplementary Figure S1. Detection of inflammatory cell infiltration and demyelination in the spinal cord of $PLP_{139–151}$-$EAE_{SJL/J}$ mice. A H&E and **B** MBP staining of cervical, thoracic, and lumbar spinal cord tissues in EAE model mice during the acute episode, corresponding remission period, the second relapse, and the subsequent remission period , with the entire spinal cord tissue section displayed.

Supplementary Figure S2.

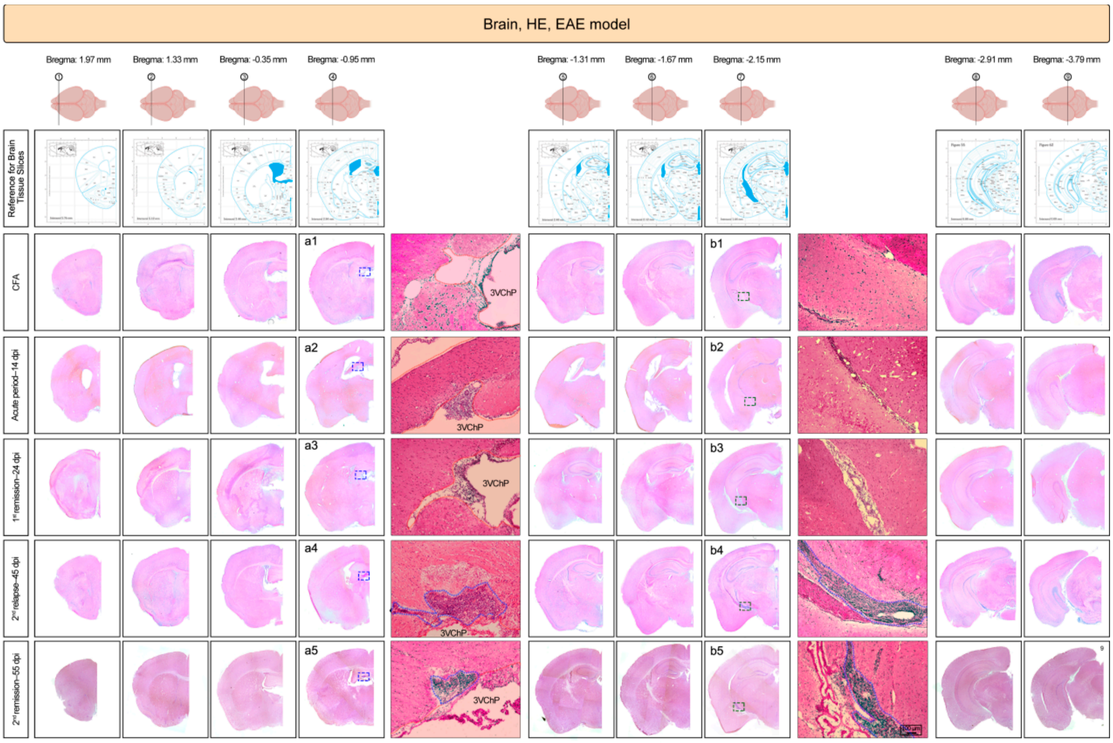

Supplementary Figure S2. Detection of inflammatory cell infiltration in brain tissues of the $PLP_{139–151}$-$EAE_{SJL/J}$ mice at different stages of the disease. In EAE mice, brain tissues were collected during the acute phase, the first remission, the second relapse, and the subsequent remission phase. Nine consecutive coronal sections from the olfactory bulb to the caudal end were prepared per mouse for H&E staining. Anatomical reference was based on The Mouse Brain Library atlas and Paxinos and Franklin' s the Mouse Brain in Stereotaxic Coordinates. Specific regions exhibiting immune cell infiltration (areas a1–a5 in the 4th section and b1–b5 in the 7th section) were examined at higher magnification.

Supplementary Figure S3

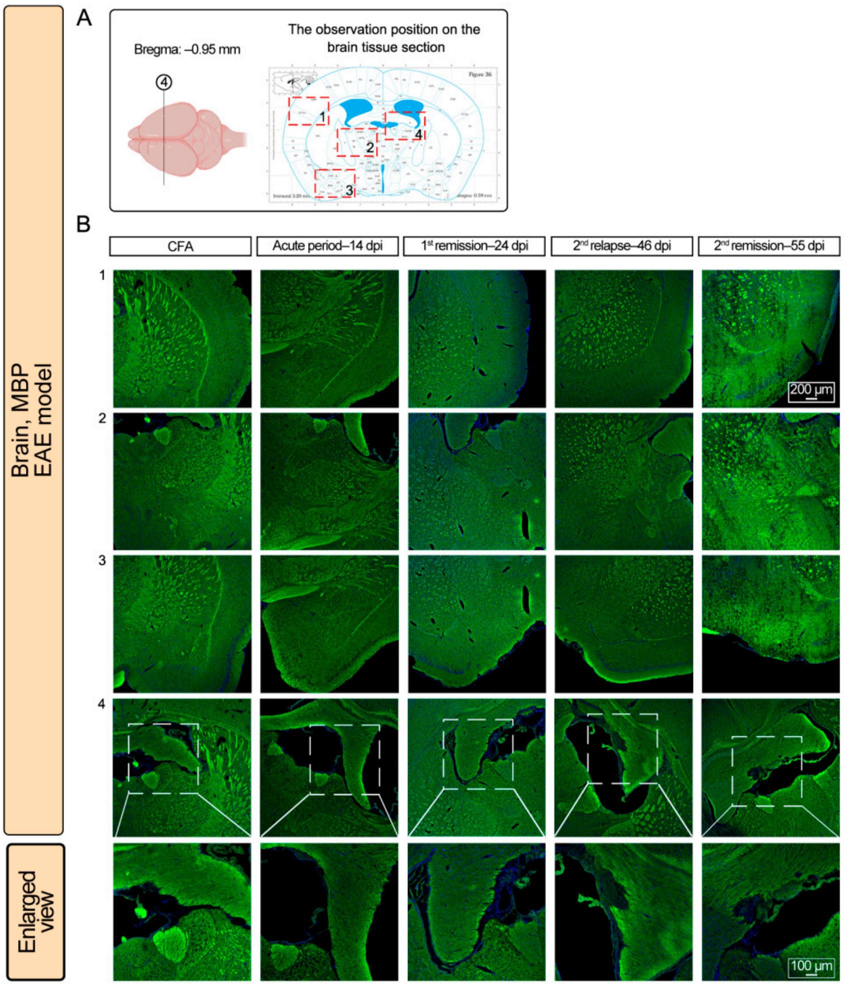

Supplementary Figure S3. Detection of demyelination in the brain of EAE model mice at different disease stages. MBP staining was performed on distinct regions within the same coronal plane of brain tissues during the acute phase, the first remission, the second relapse, and the subsequent remission phase. **A** The four examined regions (1−4) on the brain tissue section. **B** MBP staining results for regions 1 to 4 in A, with an enlarged view of the area outlined in white within region 4. Scale bars: 200 μm (overviews), 100 μm (enlarged view).

Supplementary Figure S4

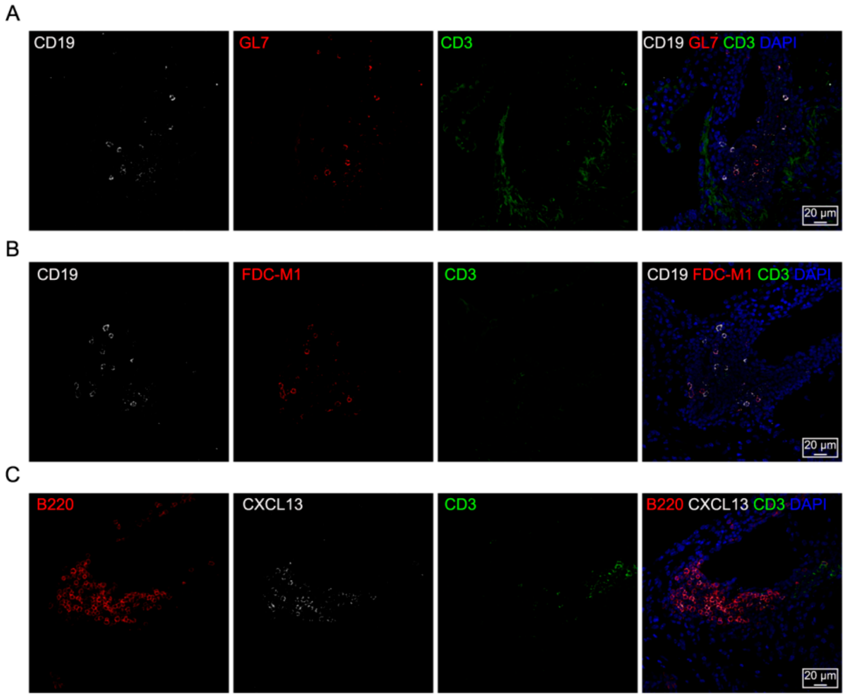

Supplementary Figure S4 Identification of TLS structures. Brain tissues were collected from mice at the second relapse peak, and serial coronal sections were obtained as comprehensively as possible. Immunofluorescence staining was then performed with the following antibodies: (A) GL7 (red), CD3 (green), CD19 (gray), and DAPI (blue). (B) FDC-M1 (red), CD3 (green), CD19 (gray), and DAPI (blue). (C) B220 (red), CD3 (green), CXCL13 (gray), and DAPI (blue). Scale bar: 20 μm.

Supplementary Figure S5

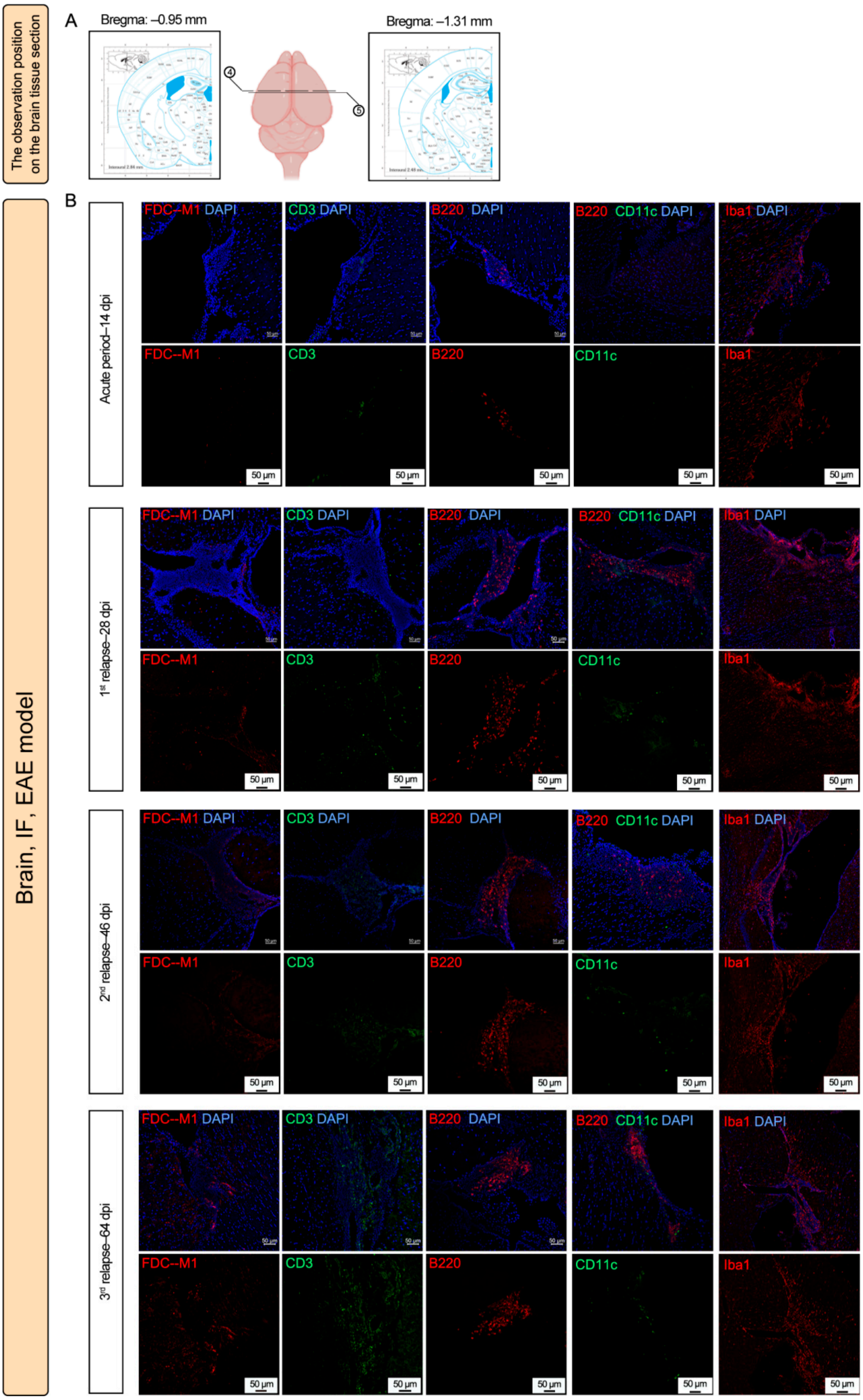

Supplementary Figure S5. Detection of immune cells involved in TLS formation in the brains of EAE model mice at different stages of the disease. Following successful induction of the EAE model, frozen sections were prepared from the brain tissues located between position 4 and position 5 (as shown in Figure S4**A**). Immunofluorescent staining was then performed on the brain TLS regions at the following disease stages: the acute episode (14 dpi), the first relapse (28 dpi), the second relapse (46 dpi), and the third relapse (64 dpi). The staining targets follicular dendritic cells (FDC-M1), T cells (CD3), **B** cells (B220), dendritic cells (CD11c), and microglia (Iba1). Scale bars: 50 μm.

Supplementary Figure S6

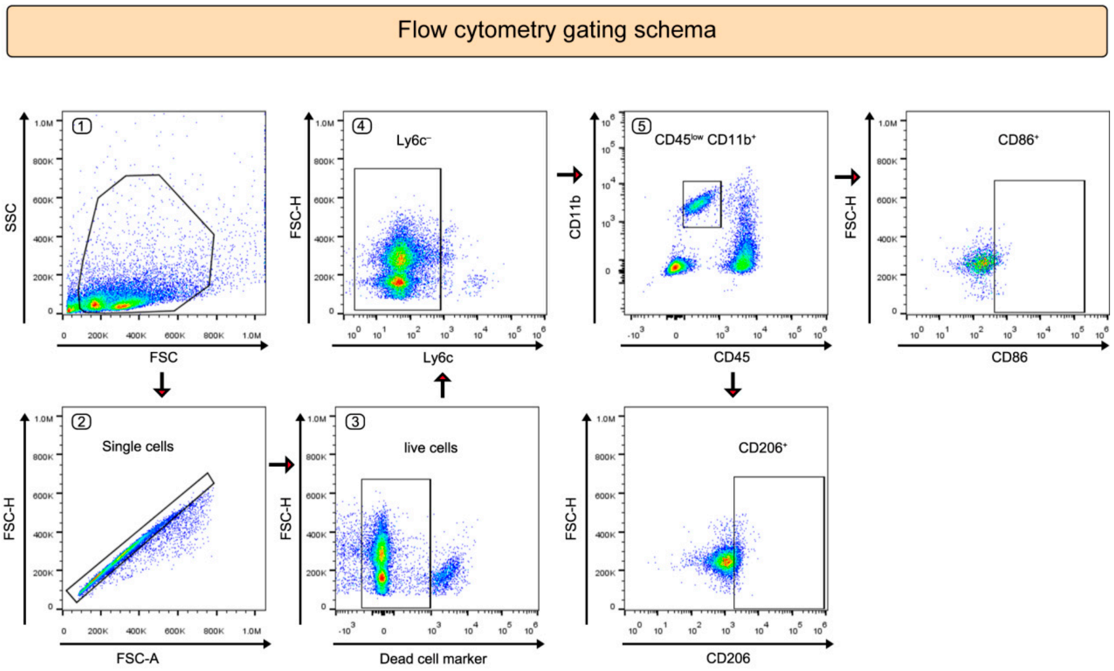

Supplementary Figure S6. Gating strategy was used to detect pro-inflammatory Ly6c^{-}CD45^{low}CD11b^{+}CD86^{+} microglia and anti-inflammatory Ly6c^{-}CD45^{low}CD11b^{+}CD206^{+} microglia in the brain of mice.

Supplementary Figure S7

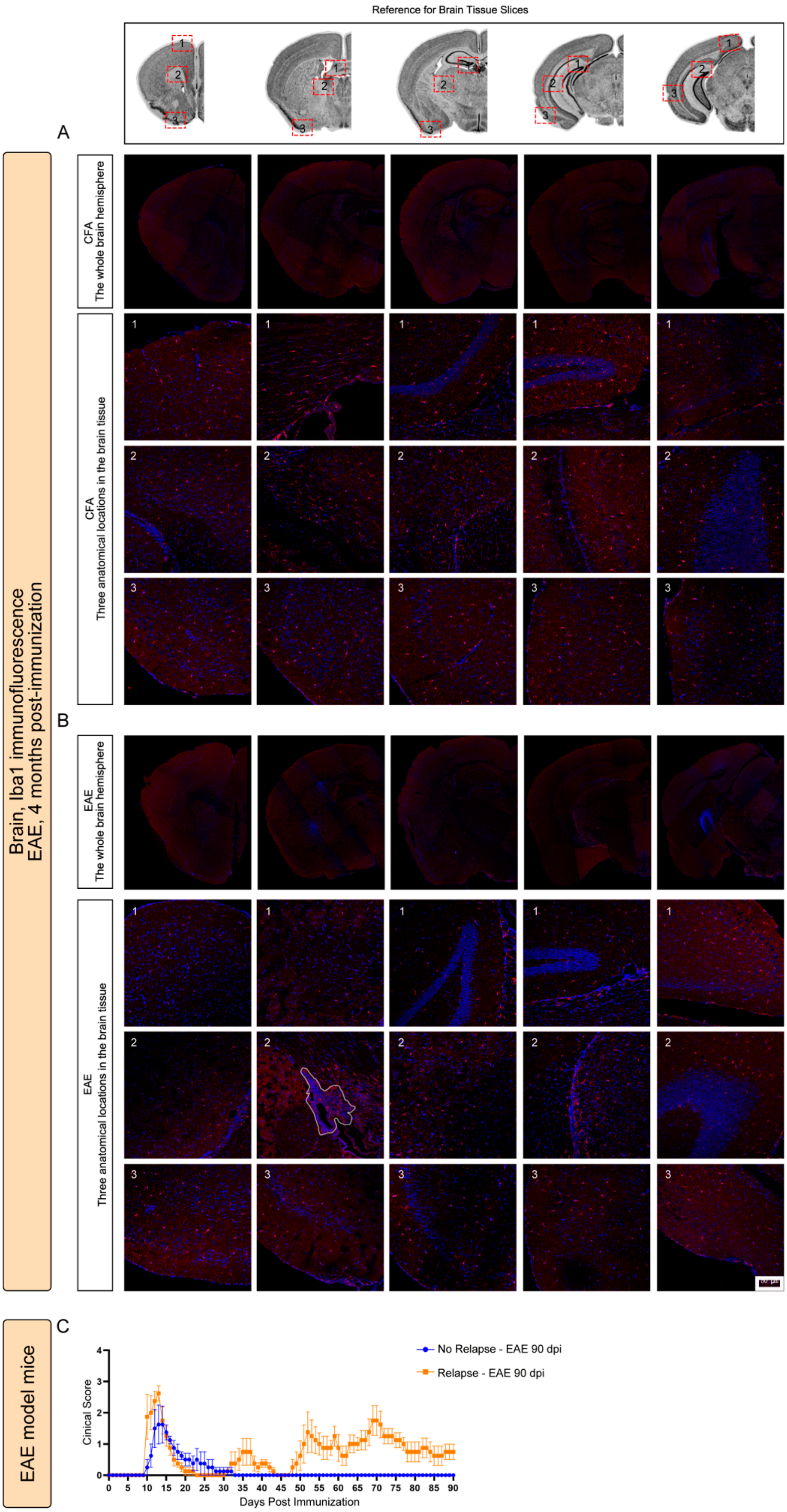

Supplementary Figure S7. Detection of Iba1$^+$ microglia in different regions of brain tissues in EAE mice. PLP$_{139–151}$-EAE$_{SJL/J}$ mice were established, at 120 dpi, brain tissues were collected for Iba1 immunofluorescent staining. For each brain sample, five consecutive sectional levels were selected, and three regions of interest (ROIs) were imaged and analyzed per level (marked with a red dashed rectangular box). Representative panoramic views and magnified images of the selected ROIs showing Iba1 staining are presented. **A** Brain sections of the CFA control mice. **B** Brain sections of the EAE model mice. **C** Establish PLP$_{139–151}$-EAE$_{SJL/J}$ model, clinical disease course of EAE mice over a 90–day observation period. Mice exhibiting a monophasic disease course (a single episode, <5% of total) were pooled and their score trajectories plotted against those of mice with a typical relapsing-remitting course (control group). n = 4.

Supplementary Figure S8

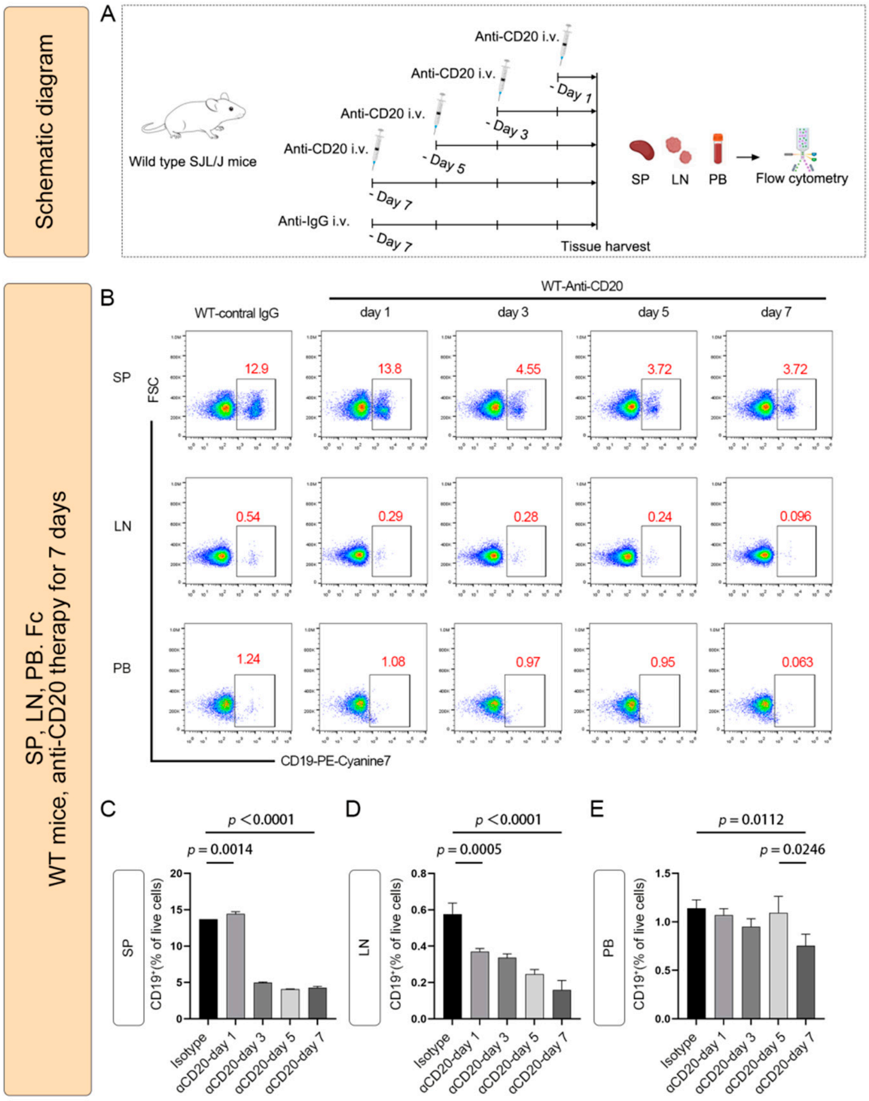

Supplementary Figure S8. Quality Control of αCD20 Antibody. Wild-type (WT) mice were divided into five groups: Mice were administered a single dose of antibody and assessed at the time of tissue collection. The timing of administration prior to collection was varied: Group 1, 2, 3, and 4 received the αCD20 antibody 1, 3, 5, and 7 days before collection, respectively. The control group received a control antibody 7 days before. Spleens, lymph nodes, and peripheral blood were collected from all five groups to assess the B-cell depletion efficacy of the αCD20 antibody. **A** Schematic

diagram. **B** Representative flow cytometry scatter plots. **C** Quantification of $CD19^+$ B-cell levels by flow cytometry.

Supplementary Figure S9

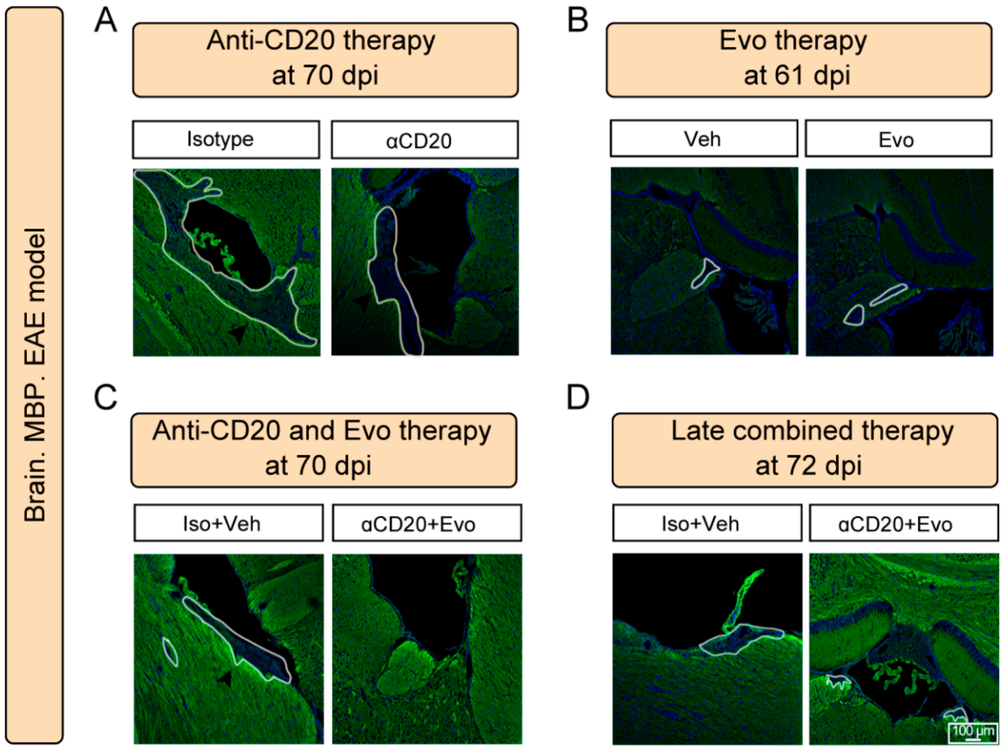

Supplementary Figure S9. Effects of αCD20 antibody, Evo, combined therapy, and late combined therapy on demyelination in the brain of EAE mice. Brain tissues were collected at the indicated time points for MBP immunofluorescence staining. **A** αCD20 antibody group and control group (70 dpi). **B** Evo group and control group (61 dpi). **C** Combined therapy group and control group (70 dpi). **D** Late combined therapy group and control group (72 dpi). White boxes indicate areas with reduced MBP staining intensity. Scale bars: 100 µm.

Supplementary Figure S10

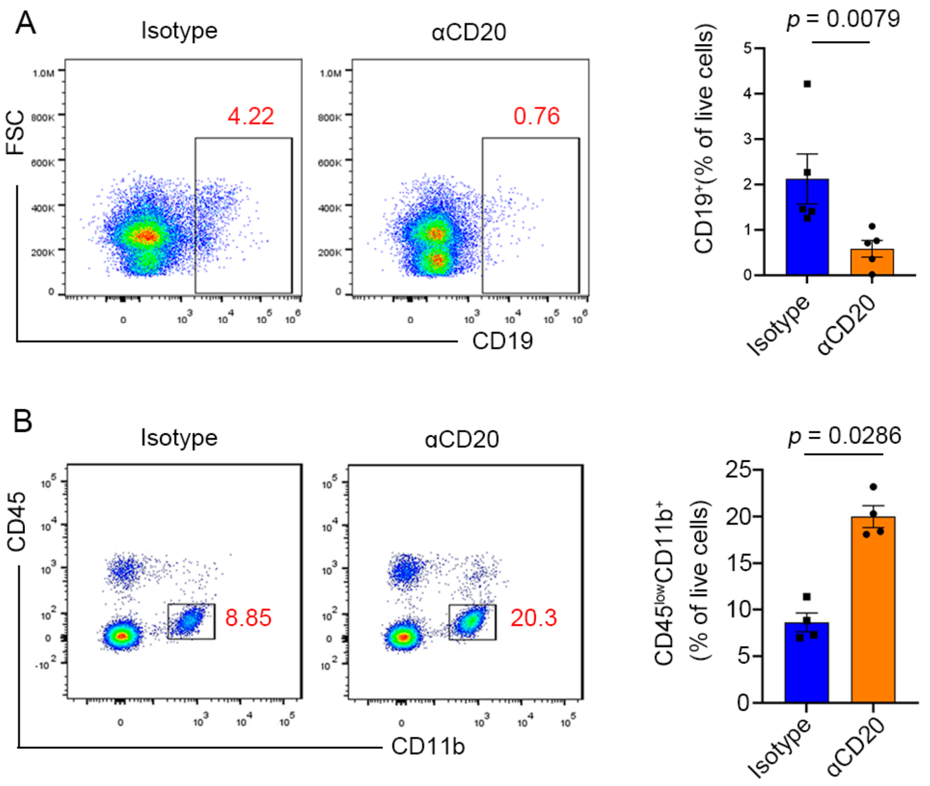

Supplementary Figure S10 Flow cytometric analysis of microglial cells and CD19$^+$ B cells in mice treated with αCD20 antibody or isotype control. (A) Proportion anti-inflammatory CD45^{low}CD11b$^+$ microglia. Mean values ± SEM. n = 4. Mann-Whitney test. The Hodges–Lehmann estimate of the median difference was 0.81 (97.14% CI: 0.2700 to 1.080). (B) Proportion anti-inflammatory CD19$^+$ B cells. Mean values ± SEM. n = 5. Mann–Whitney test. The Hodges–Lehmann estimate of the median difference was 1.19 (96.83% CI: 0.38 to 3.51).

Supplementary Figure S11

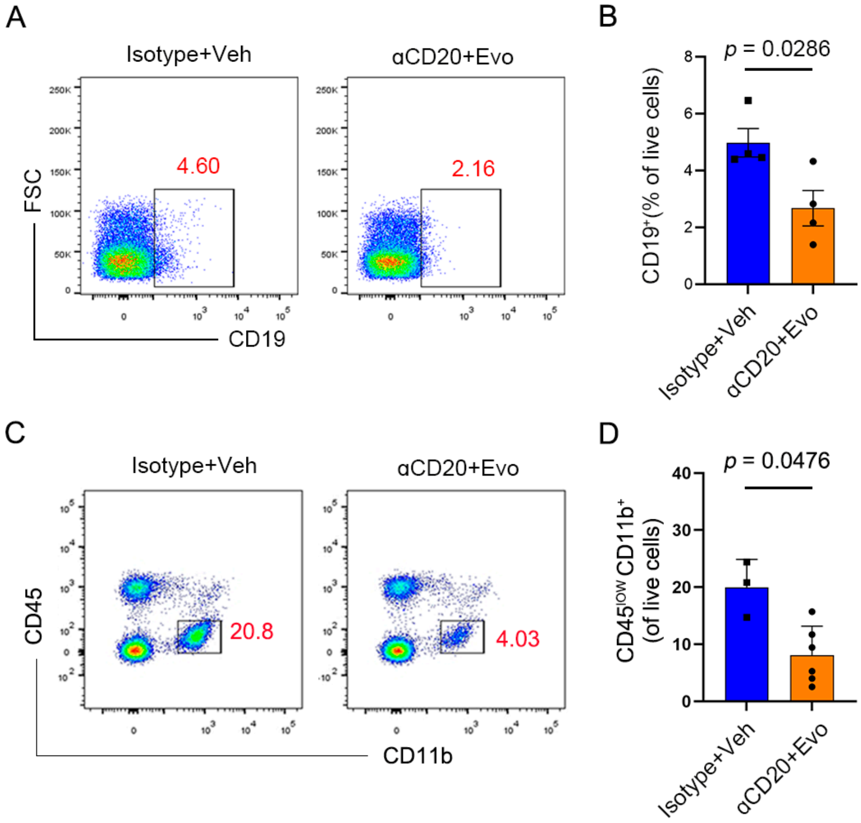

Supplementary Figure S11 Flow cytometric analysis of $CD19^+$ B cells in the brain following combined therapy and the control vehicle. (A-B)Proportion anti-inflammatory $CD19^+$ B cells. Mean values ± SEM. n = 4. Mann–Whitney test. The Hodges–Lehmann estimate of the median difference was 2.27 (97.14% CI: 0.070 to 5.07). (C-D) Proportion anti-inflammatory $CD45^{low}CD11b^+$ microglia. Mean values ± SEM. n = 4–6. Mann–Whitney test. The Hodges–Lehmann estimate of the median difference was 11.75 (95.24% CI: 3.00 to 20.37).

Supplementary Figure S12

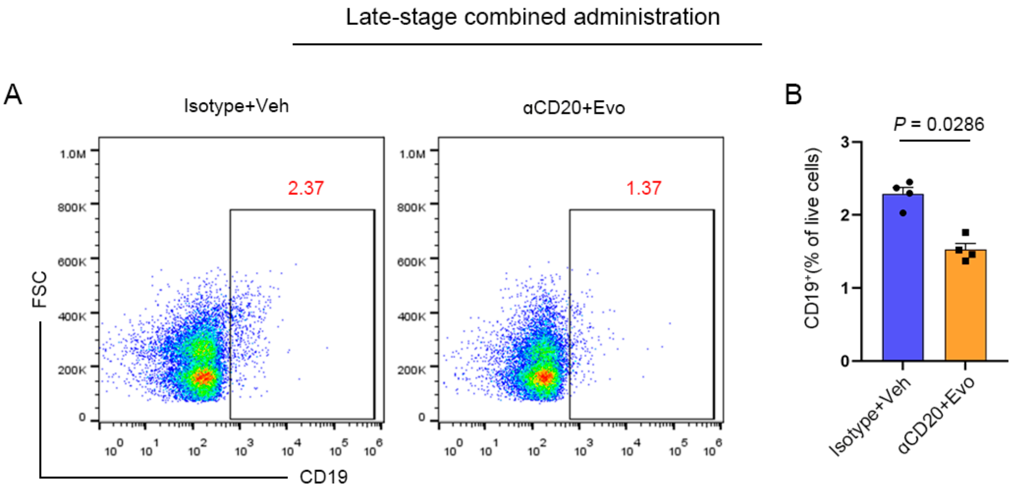

Supplementary Figure S12 Flow cytometric analysis of microglial phenotypes in the brain following late combined therapy and the control vehicle. (I) Proportion anti-inflammatory $Ly6c^{-}CD45^{low}CD11b^{+}CD206^{+}$ microglia. Mean values ± SEM. n = 4. Mann–Whitney test. The Hodges–Lehmann estimate of the median difference was 0.81 (97.14% CI: 0.2700 to 1.080).
